# Better than industry self-regulation: Compliance of mobile games with newly adopted and actively enforced loot box probability disclosure law in South Korea^☆^

**DOI:** 10.1016/j.actpsy.2025.105490

**Published:** 2025-10

**Authors:** Leon Y. Xiao, Solip Park

**Affiliations:** a School of Creative Media, City University of Hong Kong, China; b beClaws.org, London, United Kingdom; c Department of Arts and Media, School of Arts, Design and Architecture, Aalto University, Finland

**Keywords:** Loot boxes, Video games, Computer gaming regulation, Interactive entertainment and information technology law, Consumer protection, South Korea, Behavioral addiction policy

## Abstract

Loot boxes are gambling-like products inside video games that players can purchase with real-world money to obtain random rewards. Stakeholders (*e.g.*, players, parents, and policymakers) are concerned about their potential harms, *e.g.*, overspending and normalizing gambling. Recognizing that previous industry self-regulation has failed to solve the problem, South Korea started requiring companies to disclose the probabilities of getting different loot box rewards *by law* from March 2024 onwards. Content analysis found that 90 of the 100 highest-grossing iPhone games contained paid loot boxes, but only 84.4 % of those games disclosed probabilities, meaning that compliance was still not perfect. The accessibility and visual prominence of most disclosures should also be improved. South Korean regulators are actively monitoring games for compliance, including processing player complaints, and taking enforcement actions against non-compliant companies. Several companies have been fined for illegally publishing false or misleading probabilities. Other countries still solely relying on industry self-regulation should consider adopting actively enforced legal regulation instead to improve consumer protection. Behavioral addiction-related policymaking should be evidence-based. Although novel measures could justifiably be adopted based on the precautionary principle despite a lack of scientific evidence, post-implementation research on the measure's uptake and effectiveness must be conducted. Such evidence can improve policy design (including the repeal of ineffective policies) and advise other countries. Policies should be accompanied with earmarked funding for independent research assessing their efficacy; companies should be legally required to provide data; and policymakers should work with researchers before implementation to conduct longitudinal research following open science principles.

## 1 Introduction

Playing video games is now a highly popular activity worldwide: 3.2 billion of the world's eight billion population (40 %) are reportedly video game players (International Trade Administration (US), 2025). Revenue is now frequently generated not through the sale of the software but instead through players making real-world money purchases for virtual items inside video games (so-called ‘in-game purchases’), particularly on mobile phones (Zendle, Flick, Deterding, et al., 2023). One particularly controversial type of in-game purchasing is gambling-like loot boxes, which can be bought with real-world money to obtain *random* rewards (Nielsen & Grabarczyk, 2019). Some loot boxes can be obtained without payment (*e.g.*, ‘earned’ through completing in-game tasks (Hodge et al., 2022; Larche et al., 2022)), but policymakers and the present study are more concerned about ‘paid’ loot boxes that involve both real-world money and the randomization of potential results. References below to ‘loot boxes’ refer to ‘paid loot boxes,’ unless otherwise specified.

Loot boxes are implemented in 2–3 % of console and PC games, according to data from the European age rating authority (PEGI; Pan-European Game Information), which is arguably an industry source (Xiao, 2023a); however, an independent academic source instead reported that the prevalence rate of loot boxes amongst the most-played PC games is 36 % on Valve Corporation's Steam platform (Zendle et al., 2020). In any case, these include some of the most popular games on those platforms, including Electronic Arts' *EA FC* series of football simulation games that was previously known as the *FIFA* series (Heffernan, 2024; Lemmens, 2022; McMahon, 2025; Xiao & Declerck, 2023); Valve Corporation's first-person online shooter *Counter-Strike: Global Offensive* (*CS:GO*) (now, *Counter-Strike 2*) (Thorhauge & Nielsen, 2021); and so-called ‘gacha’ (which is another word used by some player communities to refer to in-game purchase mechanics that involve randomization, such as loot boxes) games generally produced in East Asia, such as the Chinese game *Genshin Impact* (Blom, 2023; Woods, 2022). Amongst mobile games, the prevalence rate of loot boxes has more recently been reported as between 80 and 90 % and therefore is much higher (Xiao, 2023b, Xiao, 2024c, Xiao, 2025a; Xiao, Henderson, & Newall, 2023; Xiao, Henderson, et al., 2024; *cf.* Zendle et al., 2020).

Critics have pointed out that loot boxes are conceptually and psychologically similar to gambling because they involve the player investing money on random and unknown outcomes (Drummond & Sauer, 2018): most of the time, the player will in fact lose money by failing to obtain a rare, random reward that they desired (Xiao, 2021a). Loot box spending has been consistently linked to problem gambling in multiple regions of the world (Close et al., 2021; Garea et al., 2021; Spicer et al., 2022; Xiao, Fraser, et al., 2024; Zendle & Cairns, 2018) and amongst both adults and adolescent samples (González-Cabrera et al., 2022; Zendle et al., 2019), which suggests that a potentially vulnerable group of consumers (*e.g.*, those experiencing gambling harms) may experience more harm from loot boxes through overspending. Loot box engagement has also been linked to excessive participation in video gaming (Garea et al., 2021; Li et al., 2019; Spicer et al., 2022), obsessive-compulsive symptoms and hoarding (Garea et al., 2023), and autistic characteristics (Charnock et al., 2024).

However, any causal links between loot box spending and worse mental health outcomes (Etchells et al., 2022; Xiao, Fraser, et al., 2024; *cf.* Drummond, Hall and Sauer, 2022, Drummond, Hall, Lowe-Calverley, Garrett and Sauer, 2025) are less clear. Over-engagement with loot boxes may represent a novel behavioral addiction on its own (Brooks & Clark, 2019); alternatively, high spending on loot boxes may represent a symptom of gambling or video gaming disorder or other negative life experiences (Xiao & Brooks, 2024). Surveys by Western European governments have consistently reported that over 20 % of teenagers spent money on loot boxes (Department for Communities (Northern Ireland), 2023; Dirección General de Ordenación del Juego [Directorate General for the Regulation of Gambling] (DGOJ) (Spain), 2023; UK Gambling Commission, 2019), and this is another point of concern because under-18s generally are restricted from accessing traditional gambling even in countries where commercial gambling has been legalized. In contrast, over 90 % of iOS and Android games with loot boxes bore advisory age ratings stating that they are suitable for children aged 12+ (Zendle et al., 2020). More recent longitudinal studies have also found that young people who spent money on loot boxes were more likely to participate in and spend more money on traditional gambling six months later (Brooks & Clark, 2022; González-Cabrera et al., 2023; Palmer et al., 2025). Comprehensive reviews of the emerging psychology literature in relation to loot boxes has been presented elsewhere (Montiel et al., 2022; Xiao, 2024a; Xiao & Brooks, 2024; Yokomitsu et al., 2021).

Concerned about players, especially children and young people, experiencing financial harm and possibly developing gambling problems through the normalization of gambling (Mills et al., 2024a), several countries have considered and tried a variety of regulatory approaches (Leahy, 2022; Moshirnia, 2018; Xiao, 2021b; Xiao, Henderson, Nielsen, & Newall, 2022). Most restrictively, Belgium attempted to ‘ban’ loot boxes by enforcing pre-existing gambling law that is uniquely widely inclusive (Belgische Kansspelcommissie [Belgian Gaming Commission], 2018), but this has not been enforced in practice due to the regulator lacking resources meaning that 82 % of the 100 highest grossing iPhone games still sold illegal loot boxes in 2022 (Xiao, 2023b). In contrast, many countries have not imposed any regulations (*e.g.*, the US adopted no laws and only recently enforced consumer law against one single company (Xiao, 2024b; *cf.* Federal Trade Commission (US), 2025)) or are relying on the video game industry to try (but fail) to self-regulate its own behaviors (*e.g.*, the UK (Department for Culture, Media, & Sport (UK), 2023; Department for Digital, Culture, Media, & Sport (UK), 2022; Ukie (UK Interactive Entertainment), 2023; Xiao & Lund, 2025)). One middle-ground approach of adopting or enforcing some formal regulation but not overly restricting either the players' ability to purchase loot boxes or the companies' ability to sell them is to legally require companies to disclose the probabilities of getting different rewards from loot boxes. This usually means a percentage-based value that can potentially help players to better understand their chances of getting the desired reward and estimate how much money they might need to spend on average to obtain said reward. Such disclosures might encourage better financial planning and discourage irrational spending amongst players. This requirement was first implemented as law in Mainland China in 2017 (Xiao, 2024c; Xiao, Henderson, et al., 2024; 文化部 [Ministry of Culture] (PRC), 2016) and has since also been adopted in Taiwan (effective from 1 January 2023 (消費者保護處 [Consumer Protection Office] (Taiwan), 2022; 行政院消費者保護會消費者保護處 [Consumer Protection Office, Consumer Protection Committee, Executive Yuan] (Taiwan), 2022)) and South Korea (Xiao, 2024b). The European Commission (2021) and the Dutch (Autoriteit Consument & Markt [Authority for Consumers & Markets] (The Netherlands), 2020) and the Italian consumer regulators (Autorità Garante della Concorrenza e del Mercato (AGCM) [Italian Competition Authority], 2020) have also argued that probability disclosures are required under EU consumer law because this information is important for the player to make an informed purchasing decision and therefore must be provided (Leahy, 2022), and the Dutch advertising regulator (College van Beroep van de Stichting Reclame Code [Board of Appeal of the Stichting Reclame Code] (The Netherlands), 2025) has recently enforced that position.

The adoption of probability disclosure requirements as an intervention (either as a new legislative rule, as in Asian countries, or by clarifying that the enforcement of pre-existing law can achieve the same result, as in EU countries) to address behavioral addiction concerns relating to loot boxes is not evidence-based policy *per se*. The measure was not experimentally tested prior to implementation (*i.e.*, its potential benefits have not been demonstrated), and its effectiveness following implementation has also not been assessed. It was merely assumed that such a measure could enhance transparency and potentially reduce harm. Before the policy was adopted, it was not known whether these benefits would materialise, and following implementation, it is not known whether those benefits have indeed materialised in practice. Very limited *post hoc* research on Chinese players following policy implementation found that players generally appreciate the improved transparency (Wen et al., 2024). However, the vast majority of players who saw probability disclosures reported that their loot box purchasing behavior did not change as a result, with only about 20 % reporting spending less money and about 10 % reporting spending more to open more loot boxes, which might even represent a policy backfiring (Xiao, Fraser, & Newall, 2023). Players in other countries who are also affected by this policy might experience and view probability disclosures differently, but evidence-based evaluations remain lacking.

No published research has considered whether loot box probability disclosures are well-understood by players. From other risk communication domains, we know that many people have difficulty understanding percentage-based information due to limited numeracy skills and risk literacy (Garcia-Retamero & Cokely, 2017). Children, towards whom many of these games are targeted and who are more vulnerable than the average adult, might especially struggle to understand and benefit from loot box probability disclosures. Even though the potential benefits of probability disclosures remain unclear, it is important to better understand how this intervention is implemented in practice in countries that already require it in order to inform policymaking both in that country and elsewhere. For example, if the implementation is poor because companies do not comply, then consumers would not benefit even if the measure were effective in practice at reducing harm. Stricter enforcement of the rule with the aim of encouraging better compliance should then be recommended; otherwise, players and parents might be misled into a false sense of security believing that they or their children are protected from potential harms but instead experience more harm due to becoming less careful (Xiao, 2023b). We therefore present evidence from South Korea, a country that recently introduced a new policy mandating loot box probability disclosures by law, with the view of informing policymaking and implementation both domestically and internationally.

### 1.1 South Korean regulation of loot boxes

Since February 2017, loot box probability disclosures have been required in South Korea through self-regulation by the domestic industry (rather than by law) (한국게임산업협회 [Korea Association of Game Industry; K-GAMES], 2017, 한국게임산업협회 [Korea Association of Game Industry; K-GAMES], 2018). The self-regulator (which is arguably conflicted due to being influenced by the commercial interests of the domestic industry) has consistently reported a high rate of compliance of over 90 %. Any non-compliance was generally blamed on foreign companies (*e.g.*, in the US, China, and Finland): a list of non-compliant games was published every month to attempt to publicly embarrass the relevant companies into complying (*e.g.*, 한국게임산업협회 [Korea Association of Game Industry; K-GAMES], 2020a, 한국게임산업협회 [Korea Association of Game Industry; K-GAMES], 2020b, 한국게임산업협회 [Korea Association of Game Industry; K-GAMES], 2021, 한국게임산업협회 [Korea Association of Game Industry; K-GAMES], 2024). This approach has been of dubious efficacy as two US games, Electronic Arts' *Apex Legends* and Valve Corporation's *Dota 2*, and one Finnish game, Supercell's *Brawl Stars*, have been listed for many years (over five years for *Dota 2*, as it is the oldest game) meaning that, despite the public denunciations, the companies have not taken any remedial actions in all of that time. This lack of proper enforcement powers (*e.g.*, delisting the games from the relevant app stores and removing them from the national market) meant that many popular games played by many South Korean consumers were non-compliant.

In addition, although the self-regulator preferred to suggest otherwise, domestic games produced by South Korean companies were also non-compliant sometimes (including having been denounced on the name-and-shame list (K-GAMES, 2024). Importantly, in January 2024, one of the leading video game companies in South Korea, Nexon, was fined ₩11.6 billion (≈US$8.9 million at that time) by the South Korean consumer protection regulator, the Fair Trade Commission, for intentionally disclosing incorrect loot box probabilities and misleading players into spending money in *Maple Story*, as internal company documents that were disclosed during the investigation process revealed (McEvoy, 2024; Park et al., 2023). (For context, these failings occurred many years before 2024 but had to be carefully investigated and so were only punished after much time has passed.) The industry's lack of accountability led to much public outcry and mass-scale protests by players (Y. Lee & Park, 2025; Park et al., 2023). These past failures casted doubt on whether South Korea should continue to rely on industry self-regulation. A bill that would have, *inter alia*, required loot box probability disclosures by law was first proposed in the National Assembly (the country's unicameral legislature) in December 2020 (대한민국 국회 [National Assembly of the Republic of Korea], 2020). That bill did not progress, but the issue continued to be debated.

Finally, on 21 March 2023, Article 33 of the Games Industry Promotion Act of South Korea [게임산업진흥에 관한 법률] was amended to require, from 22 March 2024 (*i.e.*, one year later), that video game companies disclose loot box probabilities and comply with other related obligations set out in the relevant presidential decree. That presidential decree, the Enforcement Decree of the Game Industry Promotion Act [게임산업진흥에 관한 법률 시행령] (hereinafter, the ‘Enforcement Decree’) (문화체육관광부 [Ministry of Culture, Sports and Tourism] (MCST) (South Korea), 2024a), was amended by the separate Presidential Decree No. 34114 of 9 January 2024 to newly insert Article 19–2, which itself sets out some matters that must be disclosed and also states that other matters (such as the method by which the disclosures should be made) are set out in the newly added Appendix 3–2 of the Enforcement Decree (MCST, 2024b). The MCST also published an explanatory note entitled the ‘Guideline on the Disclosure of Probability Information for Probabilistic Item [확률형 아이템 확률 정보공개 관련 해설서]’ on 19 February 2024 (MCST, 2024c) (which was, helpfully for foreign companies, followed by its official English translation on 15 March 2024 (MCST, 2024d)), to provide further details on how companies must comply (hereinafter, the ‘Explanatory Note’).

As to enforcement, non-compliance in the sense of non-disclosure is not punishable *per se*. However, Article 38(9) of the amended Games Industry Promotion Act allows the relevant Minister to order companies to make disclosures (if they have not disclosed at all) or to correct any published disclosures (if false or inaccurate information has been provided). Non-compliance with such corrective orders is punishable under the law. Article 45(11) of the same Act sets the penalties as either imprisonment of up to two years or a fine of up to ₩20 million (≈US$14,500). This financial penalty aspect is negligible and disproportionate for large video game companies whose individual games are capable of generating billions of US$ (Rousseau, 2024).

### 1.2 Research motivations

Previous research has found that the prevalence rate of loot boxes in the UK, Belgium, the Netherlands, and presumably other Western countries is about 80 % (Xiao, 2023b, 2025a; Xiao, Henderson, & Newall, 2023), whilst the prevalence rate in Mainland China was found to be over 90 % (Xiao, 2024c; Xiao, Henderson, et al., 2024). It has therefore been suggested that there might be wider cultural acceptance of loot boxes and therefore more frequent implementation in Far East Asia (*i.e.*, China, Japan, and South Korea) (Sato et al., 2024). However, South Korea, the fourth largest video game market in the world (after the US, China, and Japan (Newzoo, 2024)) has not been specifically studied before.

Previous research has found that companies generally did not comply with loot box probability disclosures requirements well in the UK, the Netherlands, and Mainland China. In the UK, a third of games with loot boxes did not disclose probabilities at all because this is required merely by industry self-regulation (Xiao, Henderson, & Newall, 2023; Xiao & Lund, 2025). Worse still, in the Netherlands, two-thirds of games contained loot boxes with no probability disclosures (Xiao, 2025a). In Mainland China, although surface-level compliance was high (and, in fact, nearly perfect) partially due to the measure being a formal, legal requirement and not industry self-regulatory, many companies disclosed probabilities using methods that were not visually prominent and difficult for players to access (Xiao, Henderson, et al., 2024) (this issue also affected UK and Dutch disclosures that were made). However, when Mainland Chinese and indeed UK games were more carefully examined, it was discovered that, even in games that disclosed loot box probabilities for the more important loot boxes, other less important loot box mechanics often did not have probability disclosures, even though those were required as well (Xiao, 2024c; Xiao & Lund, 2025).

Accordingly, compliance with South Korean law should also be monitored and assessed. However, because of research resource limitations, checking every minute requirement concerning probability disclosures set out in the Explanatory Note (MCST, 2024c) is not practicable. Accordingly, we set out to discover the prevalence rate of loot boxes in South Korea and test certain basic loot box probability disclosure compliance requirements. To situate our findings in a global context, we also compare the found South Korean rates with known rates from other countries (*i.e.*, Mainland China (Xiao, 2024c; Xiao, Henderson, et al., 2024), the Netherlands (Xiao, 2025a), and the UK (Xiao, Henderson, & Newall, 2023; Xiao & Lund, 2025)).

The following three research questions were addressed.

Firstly, how often are loot boxes implemented in the highest-grossing iPhone games on the South Korean Apple App Store (*i.e.*, what is the prevalence rate)?

Secondly, do the highest-grossing iPhone games with loot boxes in South Korea disclose probabilities and comply with other more specific and stringent requirements, such as disclosing the individual probabilities for every possible random outcome (*i.e.*, what are the compliance rates)?

Thirdly, are the probability disclosures displayed by the highest-grossing iPhone games with loot boxes in South Korea ‘reasonably prominent’ (as defined by previous research and detailed below)?

## 2 Method

A list of the 100 highest-grossing games for the iPhone platform in South Korea on 22 March 2024 was collated through data.ai, a leading analytics company. This list formed the sample, as all listed games remained available for download from the South Korean Apple App Store during the data collection period (March–April 2024). Like several previous studies on other jurisdictions (Xiao, 2023b, Xiao, 2025a; Xiao, Henderson, et al., 2024; Xiao, Henderson, & Newall, 2023; Xiao & Lund, 2025), we focused only on mobile games downloaded through the Apple App Store due to resource constraints, although this consistency does allow us to compare the results across different countries.

Smaller video game companies with an average annual revenue of less than ₩100 million (≈US$72,000) across all of its product offerings (rather than in relation to the individual game in question) are exempt from having to comply with the loot box regulations as set out in Article 19–2(2)(3) of the Enforcement Decree (MCST, 2024a). We reasonably assumed all of the 100 highest-grossing games were operated by companies that generated significantly higher revenue than that relatively low threshold.

The following variables were measured.

• Apple age rating

**Fig. 1:**
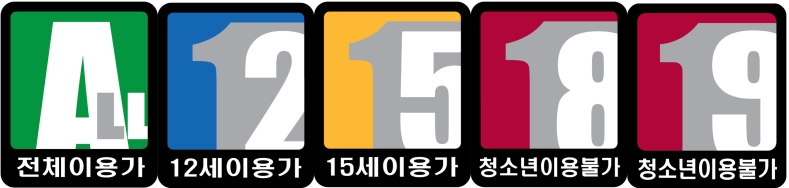
The Game Rating and Administration Committee (GRAC) All Ages, 12, 15, 18 (since defunct and replaced), and 19 rating symbols used in South Korea. © 2024 게임물관리위원회 [Game Rating and Administration Committee]

This was copied from the relevant age rating information displayed on the game's South Korean Apple App Store page. Notably, iPhone games use Apple's own proprietary age rating system and do not use the national Game Rating and Administration Committee [게임물관리위원회] (GRAC) (2024) age rating system shown in Fig. 1. The Apple App Store does not provide for an easy method by which game companies could disclose the presence of loot boxes in their games (Xiao, 2025a; Xiao & Lund, 2025); hence, we could not consistently obtain that information from an official source, unlike in relation to the Google Play Store discussed immediately below.

• Google age rating and presence of loot box presence disclosure

These were copied from the relevant age rating information displayed on the game's South Korean Google Play Store page after ensuring the parameter of ‘&gl=kr’ (that forces the International Age Rating Coalition (IARC) (2022a, 2022b) information intended for South Korea, which is based on the national GRAC age rating system, to be shown) was added to the end of the game's product listing page web address (URL; *e.g.*, https://play.google.com/store/apps/details?id=com.miHoYo.GenshinImpact&gl=kr). The Google Play Store provides the option for companies to disclose the presence of loot boxes in their games by appending a specific warning label to their product listing page (Xiao, 2023a). We collected Google Play Store data even though our study focused on the Apple App Store because it was trivially easy to assess whether the South Korean Google Play Store product listing pages for games with loot boxes were duly disclosing their presence, allowing us to advise the regulators on any needed changes and improvements. These disclosures also represented an independent source of information as to whether a game contains loot boxes that we used to inform and confirm the reliability of our assessment, which was done through gameplay.

• Presence of capsule type probabilistic items

‘Capsule type probabilistic items [캡슐형 확률형 아이템]’ are one of three categories of probabilistic items that the Explanatory Note has identified as being regulable (*e.g.*, whose probabilities must be disclosed). This category is defined as: ‘[A] probabilistic item that provides another game item when purchased or redeemed, and the type, grade, or performance of the game item provided… is determined by fortuity…’ (MCST, 2024c, p. 3). This definition includes the most traditional implementation of loot boxes, whereby a player pays real-world money to open a treasure chest to obtain random rewards (Xiao et al., 2021) and would also certainly apply to so-called ‘gacha’ summoning mechanics (*e.g.*, character, skin, and item summoning, drawing, or pulling systems) (Blom, 2023; Woods, 2022).

The Explanatory Note identified two other categories of probabilistic items. The second category relates to paying money to enhance in-game items so that they would get random upgrades (so-called ‘enhancement type probabilistic items [강화형 확률형 아이템]’ defined as ‘[a] probabilistic item that can change the [type, grade, or performance] of another game item when purchased or redeemed, and the result of the change is determined by fortuity’ (MCST, 2024c, p. 3)). The third category concerns paying money to combine different in-game items together to randomly obtain stronger items (so-called ‘combination type probabilistic items [합성형 확률형 아이템]’ defined as ‘[a] probabilistic item obtained by combining a game item, purchased directly [or] indirectly by a game product user at a cost, with another game item, and the [type, grade, or performance] of the obtained probabilistic item [is] determined by fortuity’ (MCST, 2024c, p. 3)). (This third combination type probabilistic items have previously been referred to as ‘randomized fusion mechanics’ in Mainland China (Xiao, 2022a, p. 353; 文化部 [Ministry of Culture] (PRC), 2016, para. 6).)

All three categories of probabilistic items defined in the South Korean Explanatory Note would fall within the broader definition of paid loot boxes or in-game purchases involving randomized elements that is used in other countries and by most previous academic studies (*e.g.*, Entertainment Software Rating Board (ESRB), 2020; Ukie (UK Interactive Entertainment), 2023). However, we know from previous research and gameplay experience that the second and third category of mechanics are often technically complicated and therefore difficult to be fully comprehended within a short period of gameplay (*e.g.*, one hour). Objectively assessing those other mechanics is more difficult than examining ‘capsule type probabilistic items.’ Therefore, in light of research resource constraints, we decided to leave them aside and focus on the most obvious loot box implementations, the capsule type probabilistic items as defined by the Explanatory Note (MCST, 2024c, p. 3), for the present study.

Each game was downloaded from the South Korean Apple App Store and played for up to an hour to identify whether capsule type probabilistic items were being implemented and sold in exchange for real-world money or premium in-game currency that could in turn be bought with real money. As soon as the first such mechanic has been identified in a game, coding for that game stopped regardless of how much time has passed. If no such mechanic could be found within one hour, then the game was coded as not containing capsule type probabilistic items. This method was less rigorous than spending an entire hour for each game potentially examining dozens of different loot boxes and recording their design features as adopted elsewhere (Xiao, 2024c; Xiao & Lund, 2025). However, this method has been used before and was significantly less resource intensive because many games could be coded in but a few minutes (Xiao, 2023b; Xiao, Henderson, et al., 2024; Xiao, Henderson, & Newall, 2023).

• Presence of probability disclosures

In relation to the first (and only) loot box found in each game, a corresponding probability disclosure was searched for in-game on the purchase page for the loot box. No searches elsewhere in the game (*i.e.*, beyond the loot box purchase screen) or external searches (*e.g.*, through a search engine) were conducted for disclosures that were available only on websites and not linked to from within the game because Section 2나(1) of Annex 3‐2 to the Enforcement Decree made clear that disclosures must be made on the purchase screen and any website-based disclosures, whilst permitted where the information cannot be displayed in-game due to limited space, must also be linked from the purchase screen (MCST, 2024b). All found probability disclosures were screenshotted, and the process for accessing them from the loot box purchase screen was documented. Any disclosure format, regardless of its visual prominence or ease of access, was recognized as a disclosure having been made, except with the required caveat above that the method of access for the disclosure must be knowable from the purchase page (*e.g.*, the website-based probability disclosure must be duly linked from the loot box purchase screen). Different methods of disclosure were categorized.

• Reasonable prominence of disclosures

A ‘reasonably prominent’ in-game disclosure was defined by previous research as being one that was either automatically displayed or accessible by interacting with an element that explicitly referenced ‘probabilities’ on the loot box purchase page (Xiao, Henderson, & Newall, 2023, p. 14, Xiao, Henderson, et al., 2024, p. 603). This means that the loot box purchase page should inform the player of how they can view the probabilities by making it obvious exactly which button should be interacted with (or indeed simply automatically showing them that information already on said page). Players should not be forced to try multiple ambiguous buttons on the loot box purchase page in their attempt to find the button that would lead to the probability disclosure.

• Availability of individual item-based disclosures

The different degrees of detail provided by probability disclosures can be categorized into two types: individual item-based disclosures and category-based disclosures (Xiao, 2022a, pp. 368–370). The former would require that the respective probabilities of obtaining each individual item be disclosed, whilst the latter would disclose only the probabilities for obtaining rewards of various (rarity) categories, which may each contain multiple individual rewards. It would not be possible to know the exact probabilities for any specific item if only category-based disclosures are made, and it is possible that different items that are technically within the same (rarity) category may have different probabilities, so dividing the relevant total probability for all items in the category by the number of different items in that category would not necessarily be correct or suffice. To ensure maximum transparency, Section 1가 of Annex 3‐2 to the Enforcement Decree specifically requires individual item-based disclosures to be made (MCST, 2024b). When probability disclosures were found, they were analysed as to whether they were individual item-based or not.

• Disclosure of limited availability

Certain loot boxes may be purchased ‘permanently’ (*i.e.*, at any time or at least until the online game eventually ceases operation), whilst others may only be purchased for a limited period of time or a maximum number of times. The latter types encourage players (and parents of child players) to spend money more quickly by putting time pressure on them and abusing the fear of missing out (FOMO) (Mills et al., 2024b, pp. 11–12; Nicklin et al., 2021, p. 17). Section 1라 of Annex 3‐2 to the Enforcement Decree requires companies to disclose whether the loot box is permanently or temporarily offered (*e.g.*, limited by duration or in number), and if it is the latter, to then also disclose the temporary loot box's duration of sale (*e.g.*, dates) and/or the maximum total number that will be sold as relevant (MCST, 2024b). When a loot box was found, its purchase page and probability disclosure (if any) were examined to assess whether information about said loot box's limited or permanent availability was disclosed.

• Text-searchability of webpage disclosures

Section 2나(2) of Annex 3‐2 to the Enforcement Decree also requires that any webpage disclosures must be text-searchable (MCST, 2024b). It has previously been identified that some games disclosed probability information using non-text-searchable images of tables, which are harder for players to use to quickly find specific information (*e.g.*, identify the exact probabilities for a specific reward) (Xiao, 2024c; Xiao, Henderson, et al., 2024, p. 17). The information also becomes less accessible (if at all) to readers using assistive technologies. This requirement was therefore intended to enhance accessibility and can be easily checked for compliance. When an in-game disclosure linked to a webpage disclosure, whether the content shown on that webpage was text-searchable was recorded.

• Presence of ceiling mechanics

The loot box purchase screen and any probability disclosures were also examined to identify whether the game implemented any ‘ceiling’ mechanics that guarantee a reward of a certain rarity or a specific reward will be obtained after a predetermined number of purchases have been made. These are also broadly known as ‘pity’ mechanics (Xiao, Henderson, et al., 2024, p. 5). Section 1바 of Annex 3‐2 to the Enforcement Decree specifically requires so-called ‘ceiling’ mechanics that guarantee certain rewards will be obtained, if implemented, to be disclosed alongside the conditions that must be fulfilled to activate it (MCST, 2024b). Further, the Explanatory Note requires the disclosure of additional details: namely, ‘the list of all items that can be redeemed when reaching the ceiling as well as the probability of obtaining each individual item’ (MCST, 2024c, p. 21).

• Qualification as a social casino game

Social or simulated casino games allow players to spend real-world money to purchase chips or other virtual currencies that can be used to bet on traditional gambling activities, such as playing poker, slot machines, and blackjack (Derevensky & Gainsbury, 2016; Gainsbury et al., 2014). However, importantly, these games are not recognized as actual gambling under the law in most countries (including South Korea) because there is no possibility of converting any virtual currencies (won or otherwise obtained) back into cash (Butler, 2022). Games were determined as either being a social casino game or not through gameplay using the aforementioned definition. It has been debated before whether such games should be considered as ‘containing loot boxes’ (under a broad definition) by virtue of the presence of simulated gambling mechanics inside them, such as playing blackjack or slot machines (which is obviously randomized) by spending real money without the prospect of converting any potential winning back into cash (Xiao, Henderson, & Newall, 2022; *cf.* Zendle et al., 2022). Notably, some of these games are known to also additionally implement and sell ‘traditional’ loot boxes, such as the treasure chests containing random collectible cards in *Coin Master* (Moon Active, 2015) and prize wheels offering the player the opportunity to win different prizes in *Governor of Poker 3* (Playtika, 2014), which would be ‘capsule type probabilistic items’ (see Moon Active, 2023). For the present study, the social casino mechanics themselves were not viewed as ‘loot boxes’ because they are not ‘capsule type probabilistic items’ as defined above. Instead, they were assessed as to whether they contained the latter specifically. The inclusion of this variable was not preregistered but was part of previous similar studies (Xiao, 2023b, Xiao, 2025a).

• Date and time of data collection

The date and time on and at which the game was examined were recorded.

We did not conduct formal inter-rater reliability checks because, as argued by previous research (Xiao, 2023b, p. 8, Xiao, 2023a, p. 9), this method (*i.e.*, content or game analysis to assess regulatory compliance) has become established and is known to be highly reliable. Further, all screenshots justifying the coding decisions have been reviewed by the two independent coders who discussed and agreed the results. These screenshots are also publicly shared at the data deposit link, thus allowing for wider scrutiny.

Exploratory two-sample tests of proportions that were not preregistered were conducted as detailed below under the Results section. All statistical testing was of this type.

In accordance with *Danish Code of Conduct for Research Integrity* (Ministry of Higher Education and Science (Denmark), 2014), the present study did not require research ethics assessment and approval because no human participants or personal data were involved, and only publicly available information was examined and recorded.

The present study was preregistered in the Open Science Framework at: https://doi.org/10.17605/OSF.IO/2YMJS.

## 3 Results

### 3.1 Prevalence of capsule type probabilistic items (loot boxes)

Amongst all 100 games examined through gameplay, 90 games (90.0 %) were found to have contained capsule type probabilistic items. The prevalence rates of loot boxes amongst games with various age ratings are shown in Table 1.

**Table 1:** Age ratings of, and prevalence of loot boxes amongst, the games examined (*N* = 100).

Age rating	Games with loot boxes based on coding	Games with loot boxes based on coding (cumulative)	Games with loot boxes based on self-disclosure on the Google Play Store (cumulative)
Apple 4	19 of 25 (76.0 %)	19 of 25 (76.0 %)	
Apple 9	13 of 14 (92.9 %)	32 of 39 (82.1 %)	
Apple 12	42 of 42 (100.0 %)	74 of 81 (91.4 %)	
Apple 17^a^	16 of 19 (84.2 %)	90 of 100 (90.0 %)	
GRAC All Ages	30 of 32 (93.8 %)	30 of 32 (93.8 %)	19 of 32 (59.4 %)
Google 3^b^	3 of 7 (42.9 %)	33 of 39 (84.6 %)	20 of 39 (51.3 %)
Google 7^b^	3 of 4 (75.0 %)	36 of 43 (83.7 %)	23 of 43 (53.5 %)
GRAC 12	25 of 25 (100.0 %)	61 of 68 (89.7 %)	43 of 25 (63.2 %)
GRAC 15	12 of 12 (100.0 %)	73 of 80 (91.3 %)	53 of 80 (66.3 %)
GRAC 18^c^	17 of 20 (85.0 %)	90 of 100 (90.0 %)	62 of 100 (62.0 %)

a The Apple 17 age rating was, in practice, being applied as an adult-only, 19+ age rating requiring real-life identity verification in South Korea (Apple, 2024b).

b The Google 3 and Google 7 ratings did not and still do not exist under the GRAC system; a generic number symbol was shown.

c The GRAC 18 rating has since been changed to the GRAC 19 rating.

Notably, 62 of all 100 games self-disclosed that they contained in-game purchases involving randomized elements or loot boxes (including but not limited to capsule type probabilistic items) on the Google Play Store. This meant that, on one hand, three games self-disclosed as containing loot boxes, but the present study was unable to find such a mechanic in them within one hour of gameplay. The self-disclosure should be assumed as true, meaning that the true prevalence of loot boxes is at least 93 % and potentially even higher. On the other hand, capsule type probabilistic items were found in 31 games that did not self-disclose as containing loot boxes, meaning that the loot box presence information on the Google Play Store remains unreliable. Previous research efforts have successfully demanded and caused age rating organizations to fix labelling mistakes and inaccuracies in relation to games popular in Western countries (Xiao, 2023a). However, many popular games in South Korea were only popular or even marketed in that country. This is an assumption based on, *e.g.*, the unavailability of some games except in the South Korean Apple App Store and the lack of any other in-game language options besides Korean in other games. Therefore, previous research that focused on games popular in Western countries did not have an opportunity to examine the Korean games' compliance with loot box presence labeling requirements on the Google Play Store and so could not flag them to age rating organizations for remedial actions to be taken. After being contacted by the authors with the present results and a request for a loot box presence label to be added to the Google Play Store product listing page of those 31 games with loot boxes that were not already correctly labelled, the GRAC conducted a ‘monitoring’ process and afterwards agreed and notified the authors that all these games indeed contained probabilistic items (except for one game that the GRAC could not examine on the Google Play Store). The GRAC promised to seek to have these games duly labelled as containing loot boxes.

As to the age ratings of games, Apple 17 (meaning that the content ‘may not be suitable for children under the age of 17’ (Apple, 2024a)) was displayed for many games, but this was inaccurate. In practice, the Apple 17 rating actually meant that the content was restricted to those aged 19 or above only, because, according to Apple, ‘To download or stream mature content through Apple services in South Korea, you must verify that you're at least legally 19 years old’ (Apple, 2024b). It is unclear why Apple has not introduced an Apple 19 rating instead to provide more accurate information. (After the present study concluded, Apple (2024c) started providing additional text-based advice for games rated GRAC 19, explaining their deemed unsuitability to minors in the opinion of the GRAC. However, this information would be confusingly provided alongside the Apple 17 age rating, which would still be shown. Thus, the game's Apple App Store product listing page would display two conflicting age ratings.)

On the Google Play Store, the GRAC All Ages, 12, 15, and 18 ratings shown in Fig. 1 were used. In addition, a number of games displayed the Google 3 and 7 age ratings. These two ratings did not and still do not exist under the GRAC system. Upon review on 15 July 2024, approximately three months after the initial data collection period, at least one game (Game 004 (*리니지M* [*Lineage M*])) that previously displayed GRAC 18 no longer did so and instead displayed the GRAC 19 rating: this was due to all GRAC 18 ratings having been changed to GRAC 19 on 1 July 2024 after data collection took place (GRAC, 2024d, *cf.* GRAC, 2024c). This change was due to how Article 2(10) of the Games Industry Promotion Act defined a juvenile as those under 18, but this definition was amended to being those under 19, effective from 1 January 2024 (넥슨 [Nexon], 2023). Two games (Games 008 (*EA SPORTS FC Online M*) and 015 (*브롤스타즈* [*Brawl Stars*])) that previously displayed Google 3 and 7, respectively, both continued to do so. A more comprehensive review of the Google Play Store age ratings was not conducted as the revised age ratings are not relevant to the research questions, except the facts that (i) non-GRAC age rating information was sometimes used on the Google Play Store and (ii) the games' age ratings may have been revised since data collection, both of which have been sufficiently established.

### 3.2 Probability disclosures for capsule type probabilistic items (loot boxes)

**Fig. 2:**
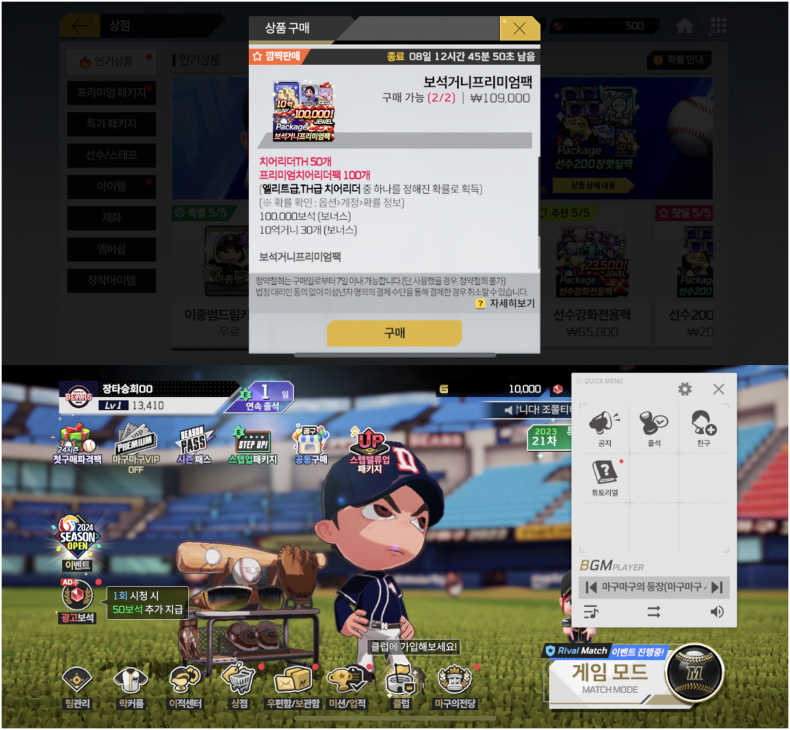
Top: A screenshot of a loot box purchase page in Game 076 (*마구마구 2024* [*MaguMagu 2024*]), displaying the text ‘확률 확인: 옵션>계정>확률 정보 [To check the probabilities: Options > Account > Probability information].’ Bottom: To follow these instructions, the player must close the loot box purchase window; navigate back to the game's home page; and locate the ‘옵션 [Options]’ button, which is obscured within the ‘QUICK MENU’ as a gear-shaped icon, and can only be tapped after the gear-shaped icon has already been tapped, as shown in the screenshot. © 2024 Netmarble.

Amongst 90 games with capsule type probabilistic items, probability disclosures were found for 76 games (84.4 %). Notably, this included one game (Game 076 (*마구마구 2024* [*MaguMagu 2024*])) that disclosed on the loot box purchase screen that the probability disclosure could be found by following a number of steps: specifically, (i) going to the ‘옵션 [Options]’ menu, (ii) then the ‘계정 [Accounts]’ menu, and (iii) finally clicking on the ‘확률 정보 [Probability information]’ button, which took the player to the relevant webpage. Accessing the Options menu required the player to exit out from the loot box purchase screen, as shown in Fig. 2. The Explanatory Note does list ‘Provide a text description on the purchase screen that explains the location where “Individual Probability Information” can be found’ as an acceptable and compliant form of disclosure where the individual circumstances of the game (*e.g.*, the hardware and UI (User Interface) design limitations) so require (MCST, 2024c, p. 29). The disclosure webpage should have been linked directly from the loot box purchase page with a button: there were no circumstances that would have made it difficult for the game company to do so. The individual circumstances of Game 076 arguably did not justify the use of a worse and less prominent and accessible form of disclosure.

**Fig. 3:**
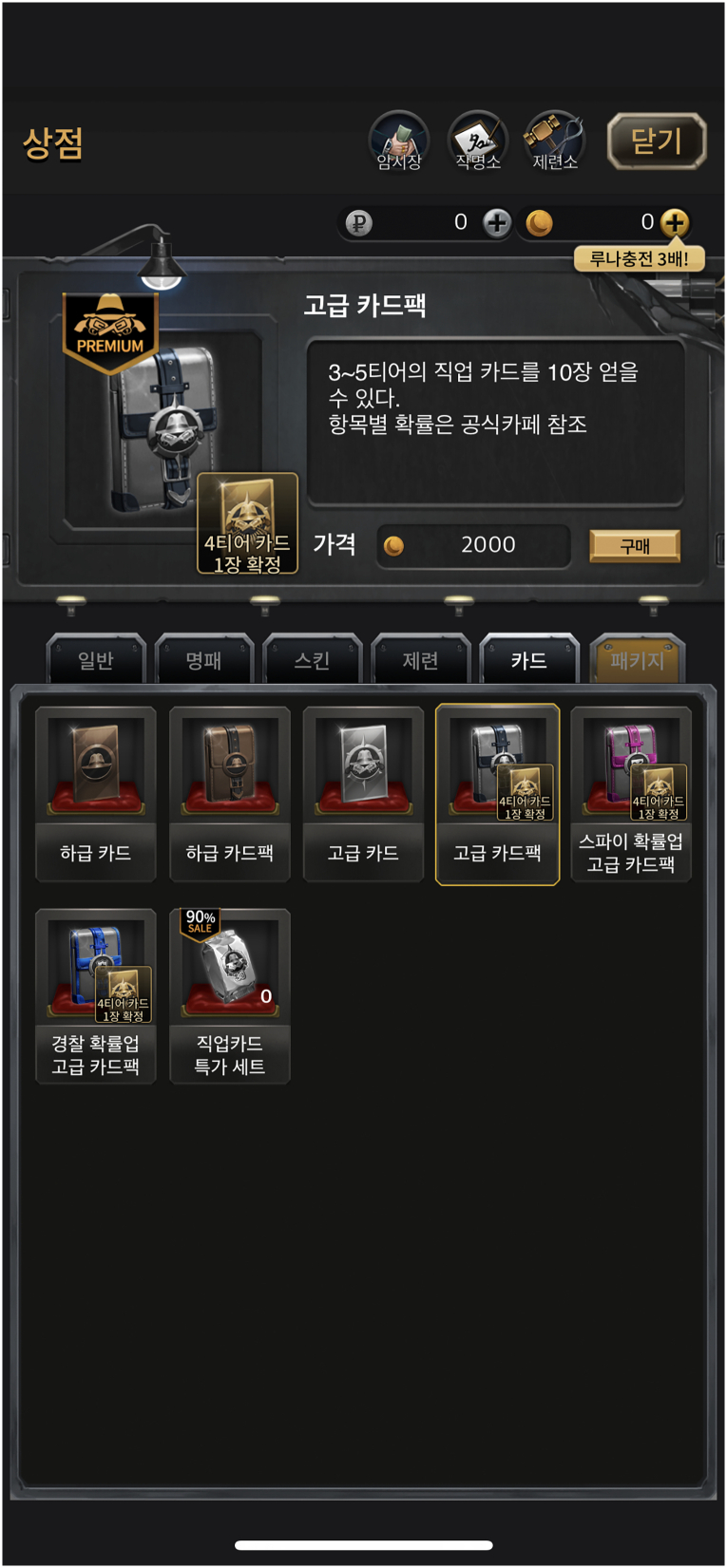
A screenshot of the loot box purchase page in Game 012 (*마피아42* [*Mafia42: Mafia Party Game*]), displaying a text description that reads, ‘항목별 확률은 공식카페 참조 [Please refer to the official cafe for the probability of each item appearing]’. © 2024 TEAM42.

Similarly, but even worse, Game 012 (*마피아42* [*Mafia42: Mafia Party Game*]) also tried to comply by stating, very vaguely, ‘항목별 확률은 공식카페 참조 [Please refer to the official cafe for the probability of each item appearing]’ without specifying the steps required to access them, as shown in Fig. 3. Relevant cultural knowledge was required to appreciate that this meant the probability disclosure is available on the game's official Naver Cafe, which is an online forum accessible *via* Naver.com—a Korean web search portal site that is similar to Google but requires substantial Korean language skills and also the completion of a Korean identity verification process to fully access. The authors confirmed that the webpage disclosure could indeed be found there as described by the in-game message. However, this disclosure was worse than Game 076's disclosure because the exact steps required for access were not provided, and the disclosure was not linked from within the game. Again, no specific circumstances could justify the company's failure to provide a button that directly linked to the relevant Naver Cafe webpage on the loot box purchase page itself: the text stating ‘공식카페 [official cafe]’ could have been hyperlinked. Nevertheless, these two games were deemed as compliant albeit quite suboptimally, following the Explanatory Note (MCST, 2024c, p. 29) and in light of the facts that (i) the information needed to access the disclosure was provided on the loot box purchase page and (ii) following those steps did cause the disclosures to be eventually shown.

**Fig. 4:**
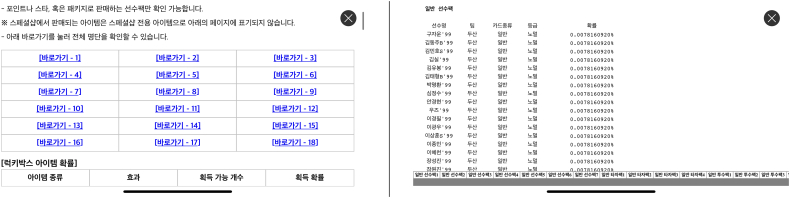
Screenshots of the probability disclosure page of Game 053 (*컴투스프로야구2024*). The game required the player to repeatedly click through different hyperlinks labelled with arbitrary numbers (left) to hopefully locate the specific probabilities they are seeking (right). Certain loot boxes' probability disclosures were not available. © 2024 TEAM42.

As to the non-compliant games, besides the ones that made no effort to comply whatsoever, certain other games, such as Game 010 (*FC 모바일* [*EA Sports FC Mobile*]) appeared to have attempted to comply with the requirement to disclose probabilities by making available a button stating ‘확률 정보 [Probability information].’ However, tapping on that button did not lead to the webpage disclosure as intended by the company because the relevant link was broken and led to an error page. This type of implementation error has previously been observed elsewhere in the Netherlands and Mainland China (Xiao, 2024c, Xiao, 2025a). Similarly, Game 053 (*컴투스프로야구2024* [*Com2uS Pro Baseball 2024*]) provided a ‘확률 [probability]’ button on the loot box purchase page for players to access the webpage disclosure that was linked to from within the game. The webpage existed but only provided probability disclosures for other loot boxes available within the game and not for the specific loot box being examined, meaning that the game was non-compliant. The webpage also displayed an overwhelming number of hyperlinks to many different loot boxes each with their own dedicated webpages and required the player to expend significant efforts to find the specific disclosure they are looking for, as shown in Fig. 4. Game 015 (*브롤스타즈* [*Brawl Stars*]) was also non-compliant because it disclosed only on its official customer support website without providing a link to the relevant webpage from within the game (*i.e.*, the loot box purchase screen specifically). The disclosure was also available only in English and not Korean, even though the game had been entirely translated to Korean.

### 3.3 Social casino games

For context, four of the 100 games (4.0 %) were deemed to be social casino games. This was a far lower prevalence rate of social casino games when compared to the 24.0 % rate found in the Netherlands in mid-2024 (*z* = −3.74, *p* < .001) (Xiao, 2025a). As mentioned, the social casino mechanics themselves were not recognized as ‘capsule type probabilistic items,’ and other in-game purchases involving randomization satisfying that definition was searched for in these games in the alternative. No such mechanics could be found in Game 089 (*WPL: 텍사스 홀덤, 토너먼트, 올인폴드, 픽앤고* [*WPL: Texas Hold'em, MTT, Sit&Go*]) (even though this game would have been deemed as containing loot boxes, broadly defined, under the methodology of previous studies (Xiao, 2023b, Xiao, 2024c, Xiao, 2025a; Xiao, Henderson, et al., 2024; Xiao, Henderson, & Newall, 2023)), whilst such mechanics were found in the other three social casino games (75.0 %). Probability disclosures were found in two of these three games (66.7 %).

### 3.4 Prevalence and probability disclosure rates compared to similar and recent results in other countries

The early 2024 South Korean capsule type probabilistic item prevalence rate of 90.0 % was not statistically significantly different from the 86.0 % loot box prevalence rate found in the Netherlands in mid-2024 by a comparable study using a near-identical methodology (*z* = 0.73, *p* = .466) (Xiao, 2025a). However, it should be caveated that a slightly more restrictive definition was used in South Korea by the present study, which reduced the prevalence rate observed (by at least 1 % because a capsule type probabilistic item could not be found in one social casino game, but that game would have been deemed as containing loot boxes if it was included in the Dutch study). Nonetheless, the studies remain broadly comparable.

The early 2024 South Korean capsule type probabilistic item disclosure rate of 84.4 % was statistically significantly higher than the 34.9 % Dutch loot box disclosure rate found in mid-2024 (*z* = 5.74, *p* < .001) (Xiao, 2025a). This held true for the rates amongst non-social casino games in both countries (85.1 % in South Korea, and 48.4 % in the Netherlands) (*z* = 4.08, *p* < .001). Because the mid-2024 Dutch disclosure rates were especially low, the overall South Korean disclosure rate was also compared to, and was found to have been significantly higher than, the mid-2021 UK rate of 64.0 % (*z* = 3.03, *p* = .001) (Xiao, Henderson, & Newall, 2023). When compared to the mid-2020 Mainland Chinese disclosure rate of 95.6 % (Xiao, Henderson, et al., 2024), the present study's rate was statistically significantly lower (*z* = −2.51, *p* = .006).

### 3.5 Method of in-game probability disclosure (prominence and accessibility)

The methods by which the 76 games disclosed probabilities are shown in Table 2. As explained in the Method section, ‘reasonably prominent’ in-game disclosure was defined by previous research as being one that was either automatically displayed or accessible by interacting with an element that explicitly referenced ‘probabilities’ on the loot box purchase page (Xiao, Henderson, & Newall, 2023, p. 14, Xiao, Henderson, et al., 2024, p. 603). The methods that were considered ‘reasonably prominent’ are marked as such in Table 2 with an asterisk. Reasonably prominent in-game disclosures were made in 31 games. These represented 34.4 % of all 90 games with capsule type probabilistic items and 40.8 % of the 76 games that disclosed probabilities in some way.

**Table 2:** Categories of observed in-game probability disclosures (*n* = 76).

Number of games (%)	Summary of disclosure method	Minimum player interactions required and whether explicit guidance was provided on the loot box purchase page (* = ‘reasonably prominent’ disclosures)
35 (46.1 %)	Immediately after tapping a small generic symbol, such as a question mark, exclamation mark, or an ‘(i)’ button, that did NOT explicitly reference probabilities	One tap, but no guidance
18 (23.7 %)	Immediately after tapping a small button explicitly referencing ‘probabilities’ or a conceptually similar term, such as a ‘확률표기 [probability disclosure]’ or a ‘확률정보 [probability information]’ button	One tap, and guidance provided*
11 (14.5 %)	After tapping a small button explicitly referencing ‘probabilities’ or a conceptually similar term as described above and then following at least one additional step, such as tapping another button	At least two taps, and guidance provided*
8 (10.5 %)	After tapping a small generic symbol as described above and then following at least one additional step, such as tapping another button	At least two taps, and no guidance
2 (2.6 %)	Automatically displayed on the loot box purchase page without requiring any additional input from the player	No tap, and no guidance needed due to automatic display*
1 (1.3 %)	By interacting with certain buttons NOT on the loot box purchase page (however, the steps required were described on the loot box purchase page and were correct when followed)	Many taps, and guidance provided
1 (1.3 %)	Not accessible from within the game, but the method required to access the disclosure externally was provided in game (and the instructions were correct when followed)	Many taps and actions external to the game required, and guidance provided

**Fig. 5:**
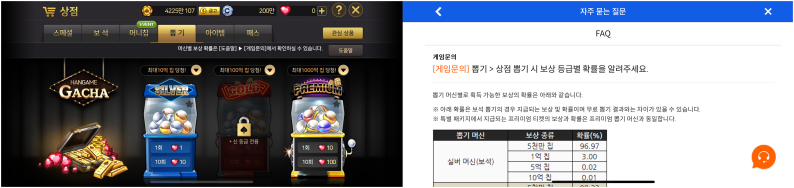
Screenshots of Game 069 (*한게임 포커*). The loot box purchase page (left) displays the ‘도움말 [Help]’ button in the upper-right corner accompanied by the text: ‘머신별 보상 확률은 [도움말] ➤ [게임문의]에서 확인하실 수 있습니다 [You can check the reward probabilities for each machine through [Help] ➤ [Questions about the Game]]’. By following this instruction, the player can access the loot box's probability disclosure (right). © 2024 NHN.

Of note, Game 069 (*한게임 포커* [*Hangame Poker*]) required the ‘도움말 [help]’ button appearing on the loot box purchase screen to be tapped and then additional steps followed for the disclosures to be displayed, as shown in Fig. 5. This is why it was coded as requiring a small generic button that did not explicitly reference probabilities to be tapped, and then other steps followed. However, additional explanatory text was provided on the loot box purchase page and described how that ‘help’ button led to the disclosure and provided the exact further steps required. Because this additional information was provided, this disclosure was arguably more prominent than the usual disclosures from the same category that required a small generic button to be tapped (and other additional steps followed) because the player knew that button would lead to the disclosure, similar to how they would know a button explicitly referencing probabilities would lead to the disclosure. However, a new category was not created to signify this minor difference. Game 069's in-game disclosure was not counted but arguably could be considered reasonably prominent as the player was informed of the relevant steps, and those steps were not particularly cumbersome when compared to, *e.g.*, Game 076's disclosure (described in Section 3.2 above), which could not be considered reasonably prominent.

### 3.6 Compliance with other specific requirements

As to the requirement to disclose the probabilities for obtaining each individual item (rather than only the probabilities for various broader categories of rewards), 54 games' disclosures were compliant, which represented 60.0 % of all 90 games with capsule type probabilistic items and 71.1 % of the 76 games that disclosed.

As to the requirement to disclose whether a loot box is available for purchase permanently or only for a limited amount of time or in limited numbers, 43 of 90 games (47.8 %) disclosed that the first capsule type probabilistic item found within that game was either permanent or limited. The other 47 games (52.2 %) did not disclose. Other relevant mechanics in these games may or may not have disclosed. In fact, it was observed in some games (such as Game 031 (*쿠키런: 킹덤* [*CookieRun: Kingdom*])) that other mechanics were advertised differently from the first mechanic encountered. Thus, the coding and the research results would have been different had the assessment been based on the other mechanics in the same game instead. However, our coding was based on the first mechanic encountered only as preregistered.

As to the requirement that any webpage disclosures must be text-searchable (rather than disclosed as a table of data shown as an image, for example), 26 games showed webpage disclosures that were linked to from in-game (or in Game 012's case, as discussed above, referred to from within the game). Amongst these, 25 webpage disclosures were text searchable (96.2 %), whilst one game's was not (3.8 %). The webpage disclosures for Game 092 (*삼국지 전략판* [*The Romance of Three Kingdoms Strategy*]) was published as images of tables of data that were far more difficult to use. The webpage disclosures for other relevant mechanics in these games may or may not have been text-searchable. In fact, it was observed in relation to Game 012 that certain other mechanics' probability disclosures were published as images of data tables and not text-searchable, unlike the disclosures for the first mechanic encountered (upon which the coding was based), thus demonstrating internal inconsistency within a single game.

As to the requirement that a ceiling mechanic must be disclosed, 47 of 90 games disclosed them, meaning that such mechanics were implemented in at least 52.2 % of games with capsule type probabilistic items (because other games that implemented them may simply have not disclosed, and other mechanics within the same game may or may not have implemented them). Amongst all 47 ceiling mechanic disclosures, only 12 (25.5 %) were compliant with the Explanatory Note's requirement to disclose a list of all potential individual items that could be obtained from the celling mechanic and their respective probabilities (MCST, 2024c, p. 21), whist the other 35 (74.5 %) were not compliant.

## 4 Discussion

### 4.1 Prevalence and compliance

The early 2024 South Korean capsule type probabilistic item prevalence rate of 90.0 % was high, but this was no longer very different from the rates now being observed in Western European countries (Xiao, 2025a), which have risen in recent years (Xiao, Henderson, & Newall, 2022; *cf.* Zendle et al., 2022). In-game purchases involving randomized aspects are undoubtedly being purchased by many players around the world, given that the vast majority of popular mobile games have prominently implemented them. Indeed, these mechanics that were found were all encountered within just one hour of gameplay and often were encountered after much less time was spent (*e.g.*, a mere few minutes after starting the game).

The observed South Korean disclosure rate of 84.4 % was better than the situation in Western European countries (*i.e.*, 34.9 % in the Netherlands and 64.0 % in the UK (Xiao, 2025a; Xiao, Henderson, & Newall, 2023)) but worse than the situation in Mainland China (*i.e.*, 95.6 % in mid-2020 and 96.9 % in early 2024 (Xiao, 2024c; Xiao, Henderson, et al., 2024)). The aforementioned studies used largely identical methods, and so their results are directly comparable. However, importantly, recent and more in-depth studies in Mainland China and the UK found that, upon closer inspection, even when games are making probability disclosures for the more obvious loot boxes (*i.e.*, the first capsule type probabilistic item encountered, which the present results are solely based upon), other less prominent loot boxes found later during gameplay often had no probability disclosures (Xiao, 2024c; Xiao & Lund, 2025). This meant that a relatively cursory examination, as was undertaken by the present study, cannot conclude that the games that were found to have disclosed in relation to the first relevant mechanic identified were in fact fully compliant with the law because other loot boxes within the same game might well have not disclosed but were not assessed herein due to a lack of resources (specifically, research time for examining games). Previous, potentially biased reports of high compliance rates of more than 90 % with the industry self-regulatory probability disclosure requirements that were adopted in South Korea in 2017 (한국게임산업협회 [Korea Association of Game Industry; K-GAMES], 2017, 한국게임산업협회 [Korea Association of Game Industry; K-GAMES], 2018), but since replaced with the legal requirements that were imposed in 2024, may have suffered from the same limitation by only examining and assessing the most obvious loot boxes (한국게임산업협회 [Korea Association of Game Industry; K-GAMES], 2020a, 한국게임산업협회 [Korea Association of Game Industry; K-GAMES], 2020b, 한국게임산업협회 [Korea Association of Game Industry; K-GAMES], 2021, 한국게임산업협회 [Korea Association of Game Industry; K-GAMES], 2024). On the other hand, if even a surface level analysis revealed obviously poor and unsatisfactory compliance as was the case in Western countries (Xiao, 2025a; Xiao, Henderson, & Newall, 2023), then the true rate of compliance would undoubtedly have been even worse.

In a similar vein, compliance was lower when the more specific and perhaps lesser-known requirements were assessed. For example, not counting the games that did not disclose at all, only 71.1 % of the disclosures found were sufficiently detailed as to provide the corresponding probabilities for each individual reward, as required by Section 1가 of Annex 3‐2 to the Enforcement Decree (MCST, 2024b). Likewise, only 12 of the 47 ceiling mechanic-related disclosures (25.5 %) provided sufficient detail as required by the Explanatory Note (MCST, 2024c, p. 21).

Companies must ensure that they comply not only in relation to the ‘main’ loot box mechanic found within their games but also with every other in-game purchasing mechanic involving randomization that are covered by the regulations. Further, companies must also ensure not only compliance with the basic requirement to disclose but also adherence to other more minor but specific requirements that are set out by policymakers. One difficulty with compliance is that companies (particularly, foreign ones) might not be aware of their regulatory obligations. It is, of course, always incumbent upon companies to be fully aware of their own legal responsibilities (and obtain local legal advice where appropriate): *ignorantia juris non excusat* [ignorance of the law is no excuse]. However, practically, policymakers and regulators could seek to improve compliance by better informing companies of their duties. To its credit, the South Korean MCST has helpfully published an official English translation (2024d) of the Explanatory Note (2024c).

Beyond merely disclosing, companies should also endeavour to maximise the visibility and accessibility of their disclosures and go above and beyond their legal obligations to be as transparent as possible with their customers. Amongst the games that disclosed probabilities, only 40.8 % used methods that were deemed reasonably prominent, which is unsatisfactory. However, it would be trivially easy for the other games to improve their disclosure method: 56.6 % of games could simply additionally mark the small generic symbol that have to be tapped to access the disclosure with explicit text referring to ‘probabilities,’ and the remaining two games (2.6 %) that disclosed the steps of how the probabilities could be accessed could simply provide a button that directly led to the relevant webpage rather than force players to undertake those steps. If these simple changes are made, then all of the in-game disclosures would be reasonably prominent.

Going forward, the standard of what is considered ‘reasonably prominent’ should also be raised to further improve consumer protection. Previous research only considered whether any guidance as to how to access the probability disclosures were provided on the loot box purchase page but did not take into account how many UI interactions are required from the player (*e.g.*, one tap, at least two taps, or even more steps (including ones external to the game), as detailed in Table 2) (Xiao, Henderson, & Newall, 2023, p. 14, Xiao, Henderson, et al., 2024, p. 603). Games 076 and 012 (detailed above), which did provide the steps required to access the probability disclosures on the loot box purchase page but required the player to perform complicated steps (such as leaving the game software entirely to search for and visit a website using a different piece of software), aptly illustrate why only accounting for whether explicit guidance was provided on the loot box purchase page is insufficient. The actual steps required should also be assessed for ease-of-use. Previous research did not consider this aspect because examples like those found in Games 076 and 012 were not encountered. Only methods that both provide explicit guidance and require one tap or even no interaction (*i.e.*, automatically displaying the probabilities) should be considered satisfactory: applying this higher standard, only 20 of 76 games' disclosures (26.3 %) met it. For all other games, beyond providing explicit guidance on the loot box purchase page as to how the disclosures could be accessed, their companies should also reduce the number of UI interactions the player is required to perform to access the probability disclosures. The legal requirement could also be tightened in a similar way to prevent companies from technically complying with the letter of the law (*e.g.*, to disclose probabilities using extremely obscure and opaque methods) but not the spirit of the law (*i.e.*, to provide consumers with *easily accessible* information about the loot box products they intend to purchase).

### 4.2 Monitoring of compliance and enforcement by the GRAC

The GRAC, whose usual work is to provide age ratings for video games in South Korea, has been put in charge of also monitoring compliance with loot box probability disclosure requirements. The GRAC held a press conference on 3 July 2024 to report on the law's implementation and both their and the MCST's monitoring and enforcement activities within the 100 days following the law taking effect (김 [Kim], 2024). The GRAC regularly monitors the 100 most popular and highest-grossing games on various platforms (*e.g.*, the Google Play Store): these games are also continuously checked for compliance when they are updated with new loot boxes. In addition, other games are monitored by the GRAC when they are reported through player complaints and by the media. Data were shared on the types of complaints received: 49 % were allegations of the probabilities having been manipulated by the company (*e.g.*, false or inaccurate disclosures); 37 % concerned probabilities not being disclosed at all; and 14 % related to other matters (such as non-compliance with the requirement to disclose loot box presence in advertising materials). It is important to recognize that older games released before the law came into force are unsurprisingly also required to comply (considering that they are still providing services and generating loot box revenue after the law became effective and often also releasing new content thereafter): it has been suggested that the regulations in other countries, such as Australia, would or should not apply retroactively to older games, but that position is unwise and untenable (Rowland, 2023; Xiao, 2023a, Xiao, 2024a, pp. 46–52).

In the 100 days since the South Korean law started being enforced, the GRAC reported monitoring 1255 cases (which, presumably, included multiple cases in relation to the same game titles) and requesting 266 corrective actions to be taken (21.2 %) (김 [Kim], 2024). About 60 % of the 266 cases related to games operated by overseas companies (presumably, *e.g.*, in China and the US), whilst 40 % related to domestic South Korean titles. Of the 266 requests, 185 of them (69.5 %) have been corrected, meaning that 81 cases have *not* been corrected yet (30.5 %). At the GRAC's request, the MCST has formally issued corrective orders in relation to five cases (or 6.2 % of the 81 non-compliant cases that remain outstanding), which all related to overseas companies. All five orders have yet to be complied with. Non-compliance with these orders could result in either imprisonment, or, more likely, a fine as detailed in Section 1.1 of the Introduction section. However, the GRAC stated that it would be difficult to apply South Korean law to overseas companies, so they will be requesting the cooperation of Google and Apple to delist these games from the South Korean national app stores and prevent them from providing services in South Korea, if the companies do not comply with the orders in due course. The GRAC also raised the interesting point that South Korean consumers are potentially harmed if a game is thusly abruptly removed following the company's non-compliance with a corrective order concerning probability disclosures because the players would lose access to in-game products and services that they have invested time and money into (see Xiao, 2024a, pp. 110–111).

The GRAC data quoted above support the present findings that there are implementation problems with the regulations. Cases of non-compliance continue to exist, and active enforcement is needed to improve compliance and consumer protection. Previously, since February 2017, loot box probability disclosures were required in South Korea by industry self-regulation only (한국게임산업협회 [Korea Association of Game Industry; K-GAMES], 2017, 한국게임산업협회 [Korea Association of Game Industry; K-GAMES], 2018). The self-regulator (which is arguably conflicted due to industry influence and funding) has always reported a high rate of compliance and generally blamed any non-compliance on foreign companies (*e.g.*, 한국게임산업협회 [Korea Association of Game Industry; K-GAMES], 2020a, 한국게임산업협회 [Korea Association of Game Industry; K-GAMES], 2020b, 한국게임산업협회 [Korea Association of Game Industry; K-GAMES], 2021, 한국게임산업협회 [Korea Association of Game Industry; K-GAMES], 2024). The fact that many problems with probability disclosures have since been identified by the GRAC (when the two sets of regulations are arguably not too dissimilar), including particularly the non-disclosure of probabilities as identified by the present study (which should not occur under either set of rules) and the widespread manipulation of probabilities by South Korean companies as identified by the Fair Trade Commission (Choi, 2025; McEvoy, 2024; Park et al., 2023), indicates that the previous industry self-regulation has not worked as effectively as the industry self-regulator would have liked to suggest. Formal legal regulation does appear more effective than industry self-regulation, which should not be surprising to any stakeholder (Xiao, 2023d, Xiao, 2023a; Xiao, Henderson, & Newall, 2023; Xiao & Lund, 2025). Although adopting legislation and appointing and funding a dedicated regulator might be costly, such public expenses may well be justified and outweighed by the reduction in harms these products cause to individuals, those around them, and society in general and the costs caused by those harms, *e.g.*, increased healthcare and public welfare needs. Other countries, such as the US and the UK, that are presently naively relying only on industry self-regulation to address concerns around loot boxes should reconsider their position.

Other countries should learn from the GRAC's active approach in both conducting regular monitoring of popular games and examining other specific games in response to player complaints. Dedicated players who have spent hundreds of hours playing a game almost certainly know far more about that game than what an employee of the regulator could glean through a few hours of gameplay (which might already be a generous estimation of how long the regulator can afford, in terms of costs and manpower, to have any single game examined). By comparison, Belgium's gambling regulator has not enforced the ‘ban’ on loot boxes at all (Xiao, 2023b); EU and Dutch regulators have not enforced consumer law against companies for loot box-related non-compliance (Xiao, 2025a); the UK consumer and advertising regulators have not effectively enforced against and prevented the continued widespread non-disclosure of the presence of loot boxes in advertising (Xiao, 2025b; Xiao, Deery, et al., 2025); and no authority has been appointed to specifically monitor and enforce compliance with the UK's industry self-regulation of loot boxes (Department for Culture, Media, & Sport (UK), 2023; Ukie (UK Interactive Entertainment), 2023; Xiao & Lund, 2025). South Korea therefore provides a blueprint for how loot box regulations could be actively enforced in practice in other countries.

Although the great progress South Korea has made should be recognized, the shortcomings of the current policy and its implementation must also be highlighted. Firstly, this process of *post hoc* corrections that the GRAC is undertaking alongside the MCST, whilst admirable, does not and cannot eliminate harm. Consumers may well have been harmed before the corrections were made. The GRAC has not discussed how players should be compensated for the money spent during the period of time when the disclosures were non-compliant: presumably, there would be no recourse (or it could only be obtained through complex and costly legal processes that render the exercise practically meaningless, unless extremely high sums were spent). Secondly, this *post hoc* process also only works if the game in question continues to operate and seeks to generate revenue over an extended period of time. Some games are intended to be released and operated only for a brief period of time, so that it can quickly generate a large amount of revenue during that time. The company does not care whether the game would be removed from the market in, hypothetically, six months' time, as it would have already made all the money it could within that period. It is unlikely that the correction process could be conducted quickly enough to prevent unscrupulous companies from unfairly profiting during that (not very) brief window. Thirdly, it is unclear whether companies, rather than individual game titles, could be banned from providing services in South Korea for repeated non-compliance. If not, then a company could release many games to make the monitoring and enforcement process difficult and costly for the GRAC and the MCST to conduct. Similarly, many corporate entities could be set up to flood the market with games that appear to have come from many different companies when in truth they came from the same commercial entity. This policy certainly works more effectively against established companies (particularly domestic ones) that are seeking to generate steady revenue over many years with their games. However, it is doubtful whether smaller overseas companies could be effectively policed even with platform involvement (*e.g.*, Apple and Google helping to prevent undesirable games from entering the South Korean market at all).

### 4.3 Definitional difficulties: what mechanics are covered by regulation?

One issue identified by the GRAC as causing difficulties for companies is that they are often unsure whether disclosures are required for certain mechanics. Specifically, this was described as the potential ambiguity between the distinction of ‘paid’ and ‘free’ loot boxes (김 [Kim], 2024). ‘Free’ mechanics are excluded from the regulations and do not have to disclose, but mechanics combining ‘free’ and ‘paid’ aspects are more difficult to deal with. Obviously, this complexity does not arise in relation to simple mechanics, such as the usual capsule type probabilistic items that the present study examined. If it costed money to open a box for random rewards, then it is a ‘paid’ mechanic. In contrast, if the box could be opened without spending any money, then it is a ‘free’ mechanic. The GRAC said that it tries to provide as much guidance on this issue as possible.

The Explanatory Note listed the following examples as not involving randomization (and by implication not falling within the regulatory ambit):

[i] Purchasing to play the game such as buying the game or subscribing to play the game;

[ii] Purchasing of an admission ticket for additional access to content that is available to anyone in the game for a limited in number of access; [and]

[iii] Time reduction item that can be purchased to reduce the time to complete a game content that is available to anyone and can normally be achieved by spending the time (MCST, 2024c, p. 2).

A FAQ (Frequently Asked Questions) document published by the GRAC (2024b, pp. 5–6) went on to answer questions regarding whether disclosures are required for specific hypothetical mechanics (hereinafter, the ‘FAQ Document’). (This FAQ Document, unlike the Explanatory Note, has not been translated to English, which should be promptly done to help non-Korean companies to better comply.) Two very similar questions asked whether a disclosure is required for loot boxes obtained from beating a dungeon that requires the purchase of an entry ticket with real-world money to attempt. The GRAC replied that if the dungeon can only be attempted by purchasing entry tickets through spending real-world money, then disclosures must be provided. However, importantly, if players can normally enter the dungeon for free a limited number of times to obtain rewards but can make more attempts by spending money to buy entry tickets, then the items obtained from the dungeon can be seen as ‘free’ and require no disclosure, irrespective of whether the attempt in question was paid or free. This is because, according to the GRAC, buying entry tickets is an in-game purchase that involves no randomization.

It is illogical to hold that no randomization was involved in this purchase simply because only an entry ticket was purchased with real-world money. The player is not merely buying that guaranteed and non-randomized entry ticket but the promise of random rewards that could potentially be obtained by using the entry ticket. It would be a legal fiction to claim that the rewards obtained through a paid entry ticket are somehow ‘free.’ In contrast, the GRAC is not disputing that loot boxes paid for with an in-game currency that is bought with real-world money (*i.e.*, so-called ‘premium’ currency) are not paid loot boxes, even though one might argue that the purchase of the currency also involved no randomization. In fact, in the FAQ Document, the GRAC (2024b, p. 5) made clear that such loot boxes bought with premium currency do in fact require disclosure for obvious reasons given most paid loot boxes are sold in this manner. It is not understood why the paid entry ticket to the dungeon is not seen as an equivalent type of premium currency used to ‘purchase’ loot boxes. The loot box (and its content) obtained from this dungeon after spending a paid entry ticket was certainly not ‘free’ and, on the contrary, was only obtained because real-world money was spent.

Concerningly, this type of loot box implementation that is allowed to evade regulation is far more complicated than the traditional loot boxes (which are opened directly) and so are naturally more difficult for consumers to understand. The inconsistent application of the regulations in South Korea is effectively encouraging companies to implement more complex and confusing loot boxes that are more likely to lead to consumer harm. Companies are being incentivized to put their loot box behind a more convoluted purchasing and opening process (*e.g.*, involving gameplay and a dungeon, as with the example in the FAQ Document (GRAC, 2024b, pp. 5–6)) because they are then somehow permitted to make no disclosures for that loot box. This aspect of the regulation should be reassessed by the MCST and the GRAC as the current interpretation is nonsensical and harms consumer rights. The Explanatory Note and the FAQ Document should be amended if deemed appropriate.

Mechanics similar to the hypothetical ones discussed were implemented in *Diablo Immortal* (Blizzard Entertainment and NetEase, 2022), and the Western and Chinese companies operating the game decided that these mechanics would constitute ‘paid loot boxes’ and therefore decided not to release the game in the Belgian and Dutch markets where, presumably, they viewed the loot box compliance risks as high (Partis, 2022). Indeed, both PEGI and the North American age rating organization (the Entertainment Software Rating Board (ESRB)) also viewed the game as containing paid loot boxes (Kennedy, 2023; Sinclair, 2023; Xiao, 2023c). This shows that the South Korean regulatory position as interpreted by the GRAC is not only internally illogical but also externally diverging from the international norm.

Importantly, neither the Explanatory Note nor the FAQ Document was helpful in clarifying whether a disclosure is required for the probabilities of obtaining different rewards from the dungeon itself (rather than the probabilities for obtaining different rewards from the loot boxes that are themselves obtained as rewards from the dungeon, which the FAQ Document addressed and stated as being not required (GRAC, 2024b, pp. 5–6), which is also an unsatisfactory regulatory position and should be revised). In other words, is the dungeon a relevant mechanic that requires disclosure? That paid entry into the dungeon certainly resulted in random rewards being obtained directly (in addition to random rewards being obtained through the loot boxes that were themselves obtained from the dungeon like a Russian doll). In summary, certain loot box implementations have been excluded from the regulatory ambit without proper justification and contrary to the text of the actual law (rather than the regulators' interpretations) and consumer interests, whilst it remains unclear whether other implementations are covered or not.

The preregistration for the present study had adopted an overly restrictive view (specifically, it was written: ‘For example, paying real-world money for more opportunities to defeat enemies in exchange for random rewards is not to be deemed as a probabilistic item in South Korea and therefore is not regulable, as the Explanatory Note explicitly stated (MCST, 2024c, p. 2)’). However, during data collection, it was deemed that there was no basis to hold that restrictive view with certainty based on the text of the actual law as explained above. The Explanatory Note could only be interpreted as having stated that buying more opportunities to defeat enemies does not *necessarily* involve randomization. It has never been addressed by the GRAC or the MCST whether the regulation would cover a mechanic that provides random rewards as a result of defeating those enemies, although the FAQ Document has stated, unsatisfactorily, that loot boxes dropped by those enemies do not require disclosure (GRAC, 2024b, pp. 5–6). In the present sample, there were three games that allowed players to spend energy to get random results. The energy could be obtained either by waiting in real-time or by spending money. These three games were coded as containing capsule type probabilistic items on the basis of these mechanics, because the energy is effectively a premium currency that is used to open loot boxes. (These three games might well have contained other capsule type probabilistic items that did not involve an energy system and would certainly have been covered by the regulations.) Depending on how South Korean law should be interpreted, these three games' identified mechanics might, unsatisfactorily, not be subject to the disclosure requirements.

### 4.4 Implications for behavioral addiction policy and research beyond South Korea

What can other countries learn from the South Korean loot box probability disclosure policy? As discussed, firstly, South Korea stopped relying on the industry to self-regulate (which has repeatedly been proven unreliable in the loot box context (Xiao, 2023a, Xiao, 2023d; Xiao, Henderson, & Newall, 2023; Xiao & Lund, 2025) and also in other behavioral addiction contexts, like gambling advertising regulation (Rossi et al., 2024)) and instead placed the policy on a legal footing with adequate deterrence powers (*i.e.*, severe punishments for non-compliance). Secondly, the rules are proactively enforced by a dedicated regulator that regularly monitors non-compliance and responds to player and media complaints, thus reinforcing the deterrence powers attached to the policy by demonstrating that they will be used and that the threats are not empty. Any future behavioral addiction policy should similarly be both (i) designed with robust deterrence measures and (ii) well-enforced.

As acknowledged in the Introduction section, even though the probability disclosure intervention has been adopted in several jurisdictions (Xiao, Henderson, et al., 2024) and widely implemented as industry self-regulation for a number of years (Xiao, Henderson, & Newall, 2023), still very little is known about its efficacy (Xiao, 2024a, pp. 115–117). That initial lack of knowledge and the imposition of this measure without having experimentally tested whether it is capable of reducing harm is potentially justified a few years ago on the precautionary principle of public health, *i.e.*, that due caution and interventions to minimize harm are justified in relation to novel technological developments even when the scientific evidence is lacking (Girela, 2006). However, this should no longer be viewed as an acceptable state of affairs. More research on behavioral addiction policy implementation must be conducted, and governments should support such efforts with not only research funding but also by enabling data access to ensure evidence-based policymaking (Ballou, 2023; Xiao, 2024a, pp. 122–125).

Alongside proactively adopting policies to address new concerns, governments should also dedicate funding towards the independent evaluations of those policies, for example, earmarking research grants that will be used to gather evidence on policy implementation to inform how that policy should be improved and whether it should be repealed depending on its efficacy (or lack thereof) in practice. Other countries could also use the evidence produced to decide whether to emulate the policy in their country. For example, Mainland China legally restricted how long young people under 18 can play video games with the aim of reducing internet addiction (Xiao, 2022b; 国家新闻出版署 [National Press and Publication Administration (PRC)], 2021). Subsequent research has sought to understand whether the measure influenced the affected Chinese young people's behavior (Yang et al., 2023; Zendle, Flick, Gordon-Petrovskaya, et al., 2023; Zhou et al., 2024), but Mainland Chinese policymakers have yet to act on the basis of such evidence. Conversely, South Korea relied on similar research to repeal restrictions on young people's video game play time that they previously adopted but were since proven ineffective at reducing internet usage and increasing sleep time through post-implementation research (Choi et al., 2018; Jo et al., 2020; C. Lee et al., 2017). Other countries might also wish to consider adopting the same preventative strategy. However, without dedicated funding, the conducting of such studies is not guaranteed, and the policy's short-term and long-term effectiveness would be unclear and become contested amongst different stakeholders with conflicting interests. Indeed, different gambling regulations aimed at reducing behavioral addiction harms have been adopted around the world. The effects of a small minority of those measures were researched (Aonso-Diego et al., 2025; García-Pérez et al., 2024; Håkansson et al., 2022; Russell et al., 2024), but many were not. Countries struggle to evaluate their own domestic policies based on research and also cannot rely on the international policy experiences of other countries to inform their own policymaking and benefit their citizens. A recent example of good practice is how the UK Government funded independent research assessing whether the industry self-regulation of loot boxes being relied upon in that country is effective or not (Department for Culture, Media, & Sport (UK), 2024).

Another important aspect where governments can uniquely intervene is to legally require companies to provide data and assist research efforts. The video gaming and gambling industries are understandably hesitant to voluntarily reveal potentially commercially sensitive data, which when used by independent researchers to assess and inform policymaking might compromise industry interests, because the results might encourage the adoption of more restrictive policies. For this reason, many policy evaluations in the behavioral addiction domain have relied on less reliable self-reported data (Yang et al., 2023; Zhou et al., 2024) or expended great efforts to negotiate industry data access with restrictions on use (Zendle, Flick, Gordon-Petrovskaya, et al., 2023) or otherwise obtained data through unconventional means, such as data scraping (Ballou et al., 2024; Xiao, 2023a, Xiao, 2025b) and data donations from players (Petrovskaya & Zendle, 2023). A recent EU law, the Digital Services Act [2022] OJ L277/1, now legally requires large social media platforms to, *inter alia*, share data on all advertising shown to EU users; this data access has enabled behavioral addiction-related policy evaluations that otherwise would have been highly difficult and costly, if not impossible, to conduct (see Xiao, 2025b; Xiao & Deery, 2025; Xiao, Deery, et al., 2025; Xiao, Petrovskaya, et al., 2025). Similar provisions could have been attached to the South Korean loot box probability disclosure law, requiring companies to share data on players' loot box spending, circulate research surveys to players containing psychometrics evaluations, and provide loot box opening results for auditing purposes. These data would have enabled easier, cheaper, and more comprehensive policy evaluations.

Indeed, following the principles of open science and in an ideal world, in addition to earmarked research funding and legally mandated data access, policymakers could collaborate with independent researchers, perhaps in consultation with the industry acknowledging possible conflicts of interest, to design and pre-register research protocols for policy evaluations *before* implementation to facilitate better quality longitudinal research (Xiao & Lund, 2025). Policymakers that introduce rules without giving researchers enough time to prepare deprive the public of the best available policy evidence. At present, that occurs frequently so we often could only conduct post-implementation policy evaluation without reference to how the situation was previously (*i.e.*, the baseline), thus limiting the researchers' ability to comment on possible causal behavioral change and policy effectiveness. Policymakers could also pre-register how they will interpret different potential research results and what policy actions they will take depending on different results to enhance transparency and be more accountable to the public (which includes the industry stakeholders, who could better prepare for different policy outcomes rather than be ambushed with a surprisingly restrictive policy that must be quickly complied with). Policymaking processes that critically self-assess the implementation of public health measures following the principles of open science would lead to more effective, transparent, and equitable behavioral addiction prevention strategies.

### 4.5 Limitations

Certain important caveats to the interpretation of the present results have already been explained above in context. Emulating previous research in other countries (Xiao, 2023b, Xiao, 2025a; Xiao, Henderson, et al., 2024; Xiao, Henderson, & Newall, 2023; Xiao & Lund, 2025), we assessed the compliance situation amongst iPhone games only. The situation amongst games on other hardware platforms (*e.g.*, PC and console games), operating systems, and app stores (*e.g.*, the Google Play Store, the Kakao Games platform, and the Samsung Galaxy Store) might vary. The definitional difference between ‘loot boxes,’ as used by previous studies (Xiao, 2023b, Xiao, 2024c, Xiao, 2025a; Xiao, Henderson, et al., 2024; Xiao, Henderson, & Newall, 2023), and ‘capsule type probabilistic items,’ as used by the present study, meant that the prevalence rates found by the present study in South Korea would not be entirely comparable to that of other countries. However, the definitional differences were unlikely to have affected the results significantly because, from the coders' personal observations and extensive experience studying loot boxes, ‘capsule type probabilistic items’ were (i) in fact the most prevalent category of loot boxes amongst the three categories identified by the Explanatory Note (MCST, 2024c, p. 3); (ii) likely significantly more prevalent than the other categories; and (iii) usually implemented in any games that also implement the other categories. Therefore, the comparisons done across different countries in the present study are justifiable. Similarly, there were also slight methodological differences when, for example, assessing the method by which a game disclosed probabilities: when multiple loot boxes were found in the same game, earlier studies coded the game based on the most prominent disclosure method found (Xiao, Henderson, et al., 2024; Xiao, Henderson, & Newall, 2023), whilst the present study based the coding on the first relevant mechanic identified only. This likely meant that the earlier results would have appeared more satisfactory than they were because the best possible result was chosen to be featured. Further, assessing only the first relevant mechanic identified likely biased the results towards showing a higher compliance rate, as more obvious loot boxes are more likely to disclose and other less obvious loot boxes within the same game might not have disclosed (Xiao, 2024c; Xiao & Lund, 2025). Similarly, our exclusive focus on ‘capsule type probabilistic items,’ similarly justified on research resource constraints, also meant that other mechanics that do fall within the ambit of regulation were not assessed as to probability disclosure compliance, thus potentially affecting the results.

The present study was conducted immediately after the regulations came into force. Although companies should have been aware for one whole year that the requirements would become effective on 22 March 2024 (because the law was amended in March 2023 but gave companies an entire year to prepare), presumably, the compliance situation immediately following the regulations' enforcement was lower than what it would become after months or even years of active enforcement as companies become more familiar with the rules (and potentially disincentivized from breaking them if enforcement proves robust). In addition, the amendment of the Enforcement Decree (MCST, 2024a), including the insertion of Annex 3‐2 (MCST, 2024b), and the publication of the Explanatory Note in Korean (MCST, 2024c) and English (MCST, 2024d) and the FAQ Document (GRAC, 2024b) all happened between January–March 2024 or within three months of the date of implementation. The official English translation of the Explanatory Note was published just one week before the regulations started being enforced, whilst the Korean-only FAQ Document was published on the day of enforcement itself. This meant that some companies may not have accessed those documents in time, and that those who did manage to access the documents might not have had sufficient time to implement the specific requirements. Make no mistake: all companies were certainly obliged to comply effectively on that date, but practically, the rather late publication of these important documents may have reduced compliance. Policymakers and regulators should in the future always ensure that any guidance documents are published with ample time for companies to process and comply.

Due to resource constraints, the present study could only examine compliance with certain aspects of the South Korean loot box regulation and could not comprehensively assess each game. Other aspects are no less important and should be evaluated by future research. The present study showed that, at least in South Korea, many of the highest-grossing games were popular in that country only: these games often did not even have non-Korean translations and were not marketed towards non-Korean players. Similar observations have been made in Mainland China (Xiao, 2024c; Xiao, Henderson, et al., 2024). This meant that the present study examined many games whose loot boxes have not previously been academically studied. In contrast, the app stores of Western countries are known to be more homogenous (or rather dominated by US companies' games (Joseph et al., 2023; Nieborg et al., 2020) and more recently, by Chinese games). The situation in different countries, especially non-Western countries, should be individually studied to improve our understanding of local markets. At present, no study has yet to be conducted in Southeast Asia, the MENA (Middle East and North Africa) region, and South America. Further research can seek to capture how these countries' app stores might be witnessing Chinese and Euro-American companies vie for dominance. Besides studying video games, another aspect of the new law is that any video game advertising must disclose the presence of loot boxes (Section 2나(3)나 of Annex 3‐2 to the Enforcement Decree (MCST, 2024b)). This is a rule that has been implemented and enforced in the UK by the advertising regulator: 93 % of UK social media advertising was previously observed as non-compliant (Advertising Standards Authority, 2024a, Advertising Standards Authority, 2024c, Advertising Standards Authority, 2024b; Xiao, 2025b). Physical and digital advertising of video games in South Korea should also be assessed for compliance. Research conducted after the present study found that only 7.6 % of social media ads for popular games with loot boxes shown to South Korean users disclosed loot box presence, and concerningly, 44.9 % of those disclosures were not sufficiently visually prominent meaning that only 4.2 % of ads were compliant (Xiao, Deery, et al., 2025). Those other regulatory rules should also be strictly enforced by the GRAC and the MCST.

In addition, as with previous research, the present study only examined whether probabilities were disclosed and whether the information provided contained the amount of detail required by law. This process is not capable of verifying whether the probability disclosures were correct and whether, more specifically, ceiling or pity mechanics operated as described in the disclosure. We did not and could not test the veracity of disclosures (including ceiling mechanic disclosures specifically) without either expending significant amounts of money purchasing loot boxes (which gives rise to ethical issue, *e.g.*, financially supporting a product that may be harmful to public health using public research funding) or accessing and auditing industry data on past loot box opening results (which is not possible for academic researchers to conduct at present). In January 2024, the South Korean consumer protection regulator, the Fair Trade Commission, fined one of the leading companies in the country, Nexon, ₩11.6 billion (≈US$8.9 million at that time) for intentionally disclosing incorrect loot box probabilities and misleading players into spending money (McEvoy, 2024; Park et al., 2023). The GRAC has stated that the Fair Trade Commission stands ready to punish companies again if they intentionally manipulate the probabilities so as to unfairly harm consumers (김 [Kim], 2024). The auditing of company documents, including all previous loot box opening results, would be necessary, and that could only be done by regulators through formal investigation processes and not by academic researchers. Since then, these threats were proven true. In April 2025, three more companies were fined for providing false or misleading loot box probabilities; two more companies were notified of alleged violations and likely fined soon thereafter; and two other companies were reportedly under investigation for possible violations (Choi, 2025). These involved well-known companies operating some of South Korea's most popular games (*e.g.*, *Ragnarok Online* (Gravity, 2002), *Lineage M* (NCSoft, 2017), *STARSEED: Asnia Trigger* (Com2uS, 2024), and *PUBG: Battlegrounds* (Krafton, 2017)), although most of the relevant violations likely occurred before the loot box probability disclosure law came into force in March 2024. Considering that the manipulation of loot box probabilities has been proven to have occurred at a large scale in South Korea, it is reasonable to suspect that these companies' failings might also have negatively affected players of the same problematic games in other countries. South Korean companies were highly unlikely to have published a version of the game with misleading probabilities in South Korea only (where they would be subject to the most regulatory scrutiny) but somehow provided a version of the game with accurate probabilities in other countries (where it would be highly unlikely for them to be scrutinsed by regulators). It is also likely that companies from other countries have committed similar unlawful acts in relation to loot box probabilities (see Autorità Garante della Concorrenza e del Mercato (AGCM) [Italian Competition Authority], 2020; College van Beroep van de Stichting Reclame Code [Board of Appeal of the Stichting Reclame Code] (The Netherlands), 2025; Federal Trade Commission (US), 2025). Regulators in other countries should promptly investigate.

## 5 Conclusion

From 22 March 2024 onwards, South Korea started formally addressing the concerns surrounding gambling-like products in video games that provide random rewards, such as loot boxes and gacha, by requiring companies to disclose the probabilities of winning different rewards by law. The present study assessed compliance with that basic requirement and a number of more specific, minor requirements. Amongst the 100 highest-grossing games, 90.0 % contained loot boxes. Disclosures were found for the first loot box mechanic encountered in 84.4 % of games containing these mechanics, which is reasonably satisfactory. However, these games likely contained other more hidden loot boxes which did not disclose probabilities that the present study did not have the opportunity to examine. Compliance with less obvious requirements, such as the need to disclose the detailed probabilities for every individual item that could potentially be obtained, was lower: only 71.1 % of games that disclosed or 60.0 % of all games with loot boxes were compliant. The video game age rating authority of South Korea (the GRAC) has been tasked with monitoring compliance and has shared details on its activities. The GRAC is actively monitoring the 100 most popular games on different platforms and spot checking on games that players have complained about (김 [Kim], 2024). As of July 2024, the GRAC has monitored 1255 cases and requested 266 corrective actions to be taken by companies, of which 185 (69.5 %) have been complied with. The GRAC has also escalated five cases to the relevant Ministry to formally issue legally binding corrective orders. It remains to be seen whether the companies involved will finally comply or be punished by law and be no longer permitted to provide services to South Korean players. The GRAC's openness to hearing from player perspectives to inform their enforcement actions should be emulated by other regulators, platforms (such as the Apple App Store and Google Play Store which also set, but does not appear to enforce, certain loot box probability disclosure rules (Gach, 2019; Kuchera, 2017; Xiao, Henderson, & Newall, 2023)), and indeed academic researchers: the players are often best placed to quickly notice and allege when games may have broken the rules (see Petrovskaya et al., 2022). Channels should be open to receive and encourage player complaints because they are the most important stakeholders as the intended beneficiary of the probability disclosure rules.

Although some implementation issues remain outstanding, South Korea is demonstrating that it is proactively addressing the loot box problem. Previously, since February 2017, the country relied on industry self-regulation. This switch to formal legal regulation has demonstrated that the previous industry self-regulatory approach was not as effective as the South Korean video game industry had vocally advertised it to have been. The law was adopted because the industry was misbehaving: domestically, many leading South Korean companies intentionally disclosed false and misleading probabilities (Choi, 2025; McEvoy, 2024; Park et al., 2023), and many overseas companies completely ignored the rules when only industry self-regulation applied. The fact that a considerable number of problems were identified since the enforcement of the law (and despite the previous implementation of, and supposedly high compliance by domestic South Korean companies with, the industry self-regulations), including what would also have been breaches of the industry self-regulation (*e.g.*, non-disclosure vis-à-vis compliance with more minor and specific requirements), suggests that the prior industry self-regulations were certainly not as effective as the current approach. Other countries (such as the US and the UK) that continue to rely solely on industry self-regulation should reevaluate, given the present evidence, whether that decision is unwise and should be revisited. Any country adopting loot box regulations and other behavioural addiction-related regulatory interventions should emulate South Korea's example: appoint and properly fund a dedicated regulator to actively monitor and enforce compliance to ensure that the regulations are effectively implemented.

## Positionality statement

L.Y.X. plays and enjoys video games and broadly views the activity very positively, except for certain aspects (*e.g.*, monetisation) that he believes should be subject to more scrutiny. In terms of L.Y.X.'s personal engagement with loot boxes, he has played and continues to play video games containing loot boxes, such as *Hearthstone* (Blizzard Entertainment, 2014) until 2018 and *Genshin Impact* (miHoYo, 2020) and *Zenless Zone Zero* (miHoYo, 2024) since their initial release. He therefore engaged and continues to engage with non-paid loot boxes on a regular basis. However, he has never purchased any loot boxes with real-world money aside from negligible spending for research purposes to, *e.g.*, confirm the presence of paid loot boxes. S.P. was born and raised in South Korea and played free-to-play video games since childhood. She is, therefore, familiar with South Korean video games containing loot boxes and randomization features as well as experiences in spending real-world money on them. This includes games such as *Blade & Soul* (NCsoft, 2012), *Kartrider* (Nexon, 2004), *Lost Ark* (Smilegate, 2018). Her loot box expenditures do not generally exceed more than ₩50,000 (roughly US$40) per month.

## CRediT authorship contribution statement

**Leon Y. Xiao:** Writing – review & editing, Writing – original draft, Visualization, Software, Resources, Project administration, Methodology, Investigation, Funding acquisition, Formal analysis, Data curation, Conceptualization, Validation. **Solip Park:** Writing – review & editing, Visualization, Investigation, Conceptualization, Validation.

## Funding information

Fieldwork for data collection by L.Y.X. was enabled by an Elite Research Travel Grant 2024 [EliteForsk-rejsestipendium 2024] awarded to L.Y.X. by the Agency for Higher Education and Science of the Danish Ministry of Higher Education and Science [Uddannelses- og Forskningsstyrelsen under Uddannelses- og Forskningsministeriet]. L.Y.X. is supported by a Presidential Assistant Professors Scheme Start-Up Research Grant (9382009) awarded by the 10.13039/100007567City University of Hong Kong [香港城市大學] (March 2025). Until November 2024, L.Y.X. was supported by a PhD Fellowship funded by the 10.13039/501100011682IT University of Copenhagen [10.13039/501100011682IT-Universitetet i København]. S.P. was primarily supported by a doctoral student grant from the 10.13039/501100002666Aalto University School of Art, Design and Architecture. She was also partially funded by the 10.13039/501100000781European Research Council-funded Ontological Reconstruction of Gaming Disorder (ORE) project (Grant agreement ID: 101042052; https://ore.jyu.fi/) as a part-time project researcher.

## Declaration of competing interest

L.Y.X. has provided paid consultancy and research services for (i) Public Group International Ltd (t/a PUBLIC) (Companies House number: 10608507), commissioned by the UK Department for Culture, Media and Sport (DCMS) to conduct independent research on understanding player experiences of loot box protections (October 2024 – May 2025); (ii) the Council of Europe International Cooperation Group on Drugs and Addiction (the Pompidou Group) on a project concerning the risks of online gambling and gaming to young people co-funded by the European Union via the Technical Support Instrument and implemented by the Council of Europe, in cooperation with the European Commission (December 2024 – May 2025); and (iii) the Institute of Public Health on a report concerning the advertising of harmful products to children funded by the Irish Department of Health and intended for its Online Health Taskforce (July 2025). L.Y.X. was employed by LiveMe, then a subsidiary of Cheetah Mobile (NYSE: CMCM), as an in-house counsel intern from July to August 2019 in Beijing, China. L.Y.X. was not involved with the monetisation of video games by Cheetah Mobile or its subsidiaries. L.Y.X. undertook a brief period of voluntary work experience at Wiggin LLP (Solicitors Regulation Authority number: 420659) in London, England, in August 2022. L.Y.X. has contributed to research projects enabled by data access provided by the video game industry, specifically Unity Technologies (NYSE:U) (October 2022 – August 2023). L.Y.X. has been invited to provide advice to the UK Department for Digital, Culture, Media and Sport and its successor (the Department for Culture, Media and Sport; DCMS) on the technical working group for loot boxes and the Video Games Research Framework. L.Y.X. was the (co-)recipient of three Academic Forum for the Study of Gambling (AFSG) postgraduate research support grants (March 2022, January 2023, and July 2024) and a minor exploratory research grant (May 2024) derived from ‘regulatory settlements applied for socially responsible purposes’ received by the UK Gambling Commission and administered by Gambling Research Exchange Ontario (GREO) and its successor (Greo Evidence Insights; Greo). L.Y.X. accepted funding to publish open-access academic papers from GREO and the AFSG that was received by the UK Gambling Commission as above (October, November, and December 2022, November 2023, and May 2024). L.Y.X. was the recipient of an Elite Research Travel Grant 2024 [EliteForsk-rejsestipendium 2024] awarded by the Agency for Higher Education and Science of the Danish Ministry of Higher Education and Science [Uddannelses-og Forskningsstyrelsen under Uddannelses-og Forskningsministeriet] (February 2024). L.Y.X. has accepted conference travel and attendance grants from the Socio-Legal Studies Association (February 2022 and February 2023); the Current Advances in Gambling Research Conference Organising Committee with support from GREO (February 2022); the International Relations Office of The Jagiellonian University (Uniwersytet Jagielloński), the Polish National Agency for Academic Exchange (NAWA; Narodowa Agencja Wymiany Akademickiej), and the Republic of Poland (Rzeczpospolita Polska) with co-financing from the European Social Fund of the European Commission of the European Union under the Knowledge Education Development Operational Programme (May 2022); the Society for the Study of Addiction (November 2022, March 2023, and November 2024); the organisers of the 13th Nordic SNSUS (Stiftelsen Nordiska Sällskapet för Upplysning om Spelberoende; the Nordic Society Foundation for Information about Problem Gambling) Conference, which received gambling industry sponsorship (January 2023); the MiSK Foundation (Prince Mohammed bin Salman bin Abdulaziz Foundation) (November 2023); and the UK Gambling Commission (March 2024). L.Y.X. has received honoraria from the Center for Ludomani for contributing parent guides about mobile games for Tjekspillet.dk, which was funded by the Danish Ministry of Health’s gambling addiction pool (Sundhedsministeriets Ludomanipulje) (March and December 2023), the Fundació Pública Tecnocampus Mataró-Maresme (TecnoCampus Mataró-Maresme Foundation) for a guest lecture (November 2023), the Young Men’s Christian Association (YMCA) of Greater Toronto Youth Gambling Awareness Program for a presentation, which was funded by the Government of Ontario, Canada (March 2024), Lunds universitet (Lund University) for the right to translate parent guides about mobile games into Swedish for Kollaspelet.se, which was funded by Mediamyndigheten (the Swedish Agency for the Media) and Barnahus Stockholm (December 2024); Shenkar College of Engineering, Design and Art for a guest lecture (December 2024); and DiGRA Korea and the Game-n-Science Institute [게임과학연구원] under the Game Culture Foundation [게임문화재단] under the Ministry of Culture, Sports and Tourism of South Korea [문화체육관광부] for participating in an academic research survey (January 2025). L.Y.X. received royalties by virtue of the copyright subsisting in some of his publications from the Authors’ Licensing and Collecting Society (ALCS) (Companies House number: 01310636) (March 2023, 2024, & 2025). A full gifts and hospitality register-equivalent for L.Y.X. is available at: https://www.leonxiao.com/about/gifts-and-hospitality-register. The up-to-date version of L.Y.X.’s conflict-of-interest statement is available at: https://www.leonxiao.com/about/conflict-of-interest. S.P. discloses no conflicts of interest.

## Data Availability

The raw data, including a full library of screenshots showing, *inter alia*, the relevant Apple App Store pages and in-game loot box purchase pages for each game, and analysis script and output are publicly available in the Open Science Framework at doi: 10.17605/OSF.IO/AUFPZ.

## References

[bb0005] Advertising Standards Authority (2024, March 20). ASA Ruling on Electronic Arts Ltd A23-1222185. https://www.asa.org.uk/rulings/electronic-arts-ltd-a23-1222185-electronic-arts-ltd.html.

[bb0010] Advertising Standards Authority (2024, March 20). ASA Ruling on Jagex Ltd A23-1216471. https://www.asa.org.uk/rulings/jagex-ltd-a23-1216471-jagex-ltd.html.

[bb0015] Advertising Standards Authority (2024, March 20). ASA Ruling on Miniclip (UK) Ltd A23-1216455. https://www.asa.org.uk/rulings/miniclip--uk--ltd-a23-1216455-miniclip--uk--ltd.html.

[bb0020] Aonso-Diego G., García-Pérez Á., Krotter A. (2025). Impact of Spanish gambling regulations on online gambling behavior and marketing strategies. Harm Reduction Journal.

[bb0025] Apple (2024). https://developer.apple.com/help/app-store-connect/reference/age-ratings.

[bb0030] Apple (2024, April 26). https://support.apple.com/en-gb/118395.

[bb0035] Apple (2024, June 18). https://developer.apple.com/news/?id=7byvco78.

[bb0040] Autorità Garante della Concorrenza e del Mercato (AGCM) [Italian Competition Authority] (2020, November 17). PS11595—Activision Blizzard—Acquisti nei videogiochi, Provvedimento n. 28452 [PS11594—Activision Blizzard—Purchases in videogames, Provision n. 28452]. https://www.agcm.it/dettaglio?tc/2025/12/&db=C12560D000291394&uid=B9FA711B7757E0B2C1258637005FA58A.

[bb0045] Autoriteit Consument & Markt [Authority for Consumers & Markets] (The Netherlands) (2020, February 11). Leidraad Bescherming online consument [Guidelines on the protection of the online consumer] (published 11 February 2020) ACM/19/035689 [Regelgeving]. https://web.archive.org/web/20200628081445/https://www.acm.nl/nl/publicaties/leidraad-bescherming-online-consument.

[bb0050] Ballou N. (2023). A manifesto for more productive psychological games research. ACM Games: Research and Practice.

[bb0055] Ballou N., Sewall C.J.R., Ratcliffe J., Zendle D., Tokarchuk L., Deterding S. (2024). Registered report evidence suggests no relationship between objectively tracked video game playtime and well-being over 3 months. Technology, Mind, and Behavior.

[bb0060] Belgische Kansspelcommissie [Belgian Gaming Commission] (2018). Onderzoeksrapport loot boxen [Research Report on Loot Boxes]. https://web.archive.org/web/20200414184710/https://www.gamingcommission.be/opencms/export/sites/default/jhksweb_nl/documents/onderzoeksrapport-loot-boxen-final-publicatie.pdf.

[bb0065] Blom J. (2023). The Genshin Impact media mix: Free-to-play monetization from East Asia. Mechademia.

[bb0070] Brooks G.A., Clark L. (2019). Associations between loot box use, problematic gaming and gambling, and gambling-related cognitions. Addictive Behaviors.

[bb0075] Brooks G.A., Clark L. (2022). The gamblers of the future? Migration from loot boxes to gambling in a longitudinal study of young adults. Computers in Human Behavior.

[bb0080] Butler B. (2022, November 16). ‘Social casino’ apps: The games exempt from Australia’s gambling laws – Because no one can win. The Guardian.

[bb0085] Charnock T., Drummond A., Hall L.C., Sauer J.D. (2024). The associations between autistic characteristics and microtransaction spending. Scientific Reports.

[bb0090] Choi J. (2025, April 23).

[bb0095] Choi J., Cho H., Lee S., Kim J., Park E.-C. (2018). Effect of the online game shutdown policy on internet use, internet addiction, and sleeping hours in Korean adolescents. Journal of Adolescent Health.

[bb0100] Close J., Spicer S.G., Nicklin L.L., Uther M., Lloyd J., Lloyd H. (2021). Secondary analysis of loot box data: Are high-spending “whales” wealthy gamers or problem gamblers?. Addictive Behaviors.

[bb0105] College van Beroep van de Stichting Reclame Code [Board of Appeal of the Stichting Reclame Code] (The Netherlands) (2025, April 15). Xiao v MY.GAMES 2024/00251. https://www.reclamecode.nl/uitspraak/?uitspraakId=504990.

[bb0110] Department for Communities (Northern Ireland) (2023, December 6). Experience of gambling by young people in Northern Ireland: Findings from the Young Persons' Behaviour and Attitudes Survey 2022. https://datavis.nisra.gov.uk/communities/experience-of-gambling-by-young-people-in-northern-ireland-2022.html.

[bb0115] Department for Culture, Media & Sport (UK) (2023, July 18). Loot boxes in video games: Update on improvements to industry-led protections. GOV.UK. https://www.gov.uk/guidance/loot-boxes-in-video-games-update-on-improvements-to-industry-led-protections.

[bb0120] Department for Culture, Media & Sport (UK) (2024, October 2). R&D science & analysis programme: Understanding player experiences of loot box protections. https://www.contractsfinder.service.gov.uk/notice/3dbee6bd-9f81-4729-a754-059a691e251a.

[bb0125] Department for Digital, Culture, Media & Sport (UK) (2022, July 17). Government response to the call for evidence on loot boxes in video games. GOV.UK. https://www.gov.uk/government/consultations/loot-boxes-in-video-games-call-for-evidence/outcome/government-response-to-the-call-for-evidence-on-loot-boxes-in-video-games.

[bb0130] Derevensky J.L., Gainsbury S.M. (2016). Social casino gaming and adolescents: Should we be concerned and is regulation in sight?. International Journal of Law and Psychiatry.

[bb0135] Dirección General de Ordenación del Juego [Directorate General for the Regulation of Gambling] (DGOJ) (Spain) (2023). Estudio de Prevalencia de Juego 2022–2023 [Gambling Prevalence Study 2022–2023]. https://www.ordenacionjuego.es/es/estudio-prevalencia.

[bb0140] Drummond A., Hall L.C., Lowe-Calverley E., Garrett E., Sauer J.D. (2025). Loot box spending is associated with greater distress when normalized to disposable income: A reanalysis and extension of Etchells et al. and Xiao et al.. Royal Society Open Science.

[bb0145] Drummond A., Hall L.C., Sauer J.D. (2022). Surprisingly high prevalence rates of severe psychological distress among consumers who purchase loot boxes in video games. Scientific Reports.

[bb0150] Drummond A., Sauer J.D. (2018). Video game loot boxes are psychologically akin to gambling. Nature Human Behaviour.

[bb0155] Entertainment Software Rating Board (ESRB) (2020, April 13). Introducing a new interactive element: In-game purchases (includes random items). ESRB Official Website.

[bb0160] Etchells P.J., Morgan A.L., Quintana D.S. (2022). Loot box spending is associated with problem gambling but not mental wellbeing. Royal Society Open Science.

[bb0165] European Commission (2021, December 29). Commission Notice – Guidance on the interpretation and application of Directive 2005/29/EC of the European Parliament and of the Council concerning unfair business-to-consumer commercial practices in the internal market (C/2021/9320) [2021] OJ C526/1. https://eur-lex.europa.eu/legal-content/EN/TXT/?uri=CELEX:52021XC1229(05).

[bb0170] Federal Trade Commission (US) (2025, January 17). https://www.ftc.gov/news-events/news/press-releases/2025/01/genshin-impact-game-developer-will-be-banned-selling-lootboxes-teens-under-16-without-parental.

[bb0175] Gach E. (2019, May 30). Google now requires app makers to disclose loot box odds. https://kotaku.com/google-now-requires-app-makers-to-disclose-loot-box-odd-1835134642.

[bb0180] Gainsbury S.M., Hing N., Delfabbro P.H., King D.L. (2014). A taxonomy of gambling and casino games via social media and online technologies. International Gambling Studies.

[bb0185] García-Pérez Á., Krotter A., Aonso-Diego G. (2024). The impact of gambling advertising and marketing on online gambling behavior: An analysis based on Spanish data. Public Health.

[bb0190] Garcia-Retamero R., Cokely E.T. (2017). Designing visual aids that promote risk literacy: A systematic review of Health Research and evidence-based design heuristics. Human Factors.

[bb0195] Garea S.S., Drummond A., Sauer J.D., Hall L.C., Williams M.N. (2021). Meta-analysis of the relationship between problem gambling, excessive gaming and loot box spending. International Gambling Studies.

[bb0200] Garea S.S., Sauer J.D., Hall L.C., Williams M.N., Drummond A. (2023). The potential relationship between loot box spending, problem gambling, and obsessive-compulsive gamers. Journal of Behavioral Addictions.

[bb0205] Girela M.Á.R. (2006). Risk and reason in the European Union law. European Food and Feed Law Review.

[bb0210] González-Cabrera J., Basterra-González A., Montiel I., Calvete E., Pontes H.M., Machimbarrena J.M. (2022). Loot boxes in Spanish adolescents and young adults: Relationship with internet gaming disorder and online gambling disorder. Computers in Human Behavior.

[bb0215] González-Cabrera J., Basterra-González A., Ortega-Barón J., Caba-Machado V., Díaz-López A., Pontes H.M., Machimbarrena J.M. (2023). Loot box purchases and their relationship with internet gaming disorder and online gambling disorder in adolescents: A prospective study. Computers in Human Behavior.

[bb0220] Håkansson A., Sundvall A., Lyckberg A. (2022). Effects of a national preventive intervention against potential COVID-19–related gambling problems in online gamblers: Self-report survey study. JMIR Formative Research.

[bb0225] Heffernan C. (2024). ‘It’s in the game’: FIFA videogames and the misuse of history. Sport in History.

[bb0230] Hodge S.E., Vykoukal M., McAlaney J., Bush-Evans R.D., Wang R., Ali R. (2022). What’s in the box? Exploring UK players’ experiences of loot boxes in games; the conceptualisation and parallels with gambling. PLoS One.

[bb0235] International Age Rating Coalition (IARC) (2022). About the International Age Rating Coalition. https://www.globalratings.com/about.aspx.

[bb0240] International Age Rating Coalition (IARC) (2022). How the international age rating coalition works. https://www.globalratings.com/how-iarc-works.aspx.

[bb0245] International Trade Administration (US) (2025). Video games sector. https://www.trade.gov/media-entertainment-video-games-sector.

[bb0250] Jo W., Sunder S., Choi J., Trivedi M. (2020). Protecting consumers from themselves: Assessing consequences of usage restriction Laws on online game usage and spending. Marketing Science.

[bb0255] Joseph D., Nieborg D., Young C.J. (2023). One big store: Source diversity and value capture of digital games in National app store instances. International Journal of Communication.

[bb0260] Kennedy V. (2023, May 23). https://www.eurogamer.net/activision-blizzard-fined-just-5k-over-diablo-immortal-loot-boxes.

[bb0265] Kuchera B. (2017, December 21). https://www.polygon.com/2017/12/21/16805392/loot-box-odds-rules-apple-app-store.

[bb0270] Larche C.J., Chini K., Lee C., Dixon M.J. (2022). To pay or just play? Examining individual differences between purchasers and earners of loot boxes in Overwatch. Journal of Gambling Studies.

[bb0275] Leahy D. (2022). Rocking the boat: Loot boxes in online digital games, the regulatory challenge, and the EU’s Unfair Commercial Practices Directive. Journal of Consumer Policy.

[bb0280] Lee C., Kim H., Hong A. (2017). Ex-post evaluation of illegalizing juvenile online game after midnight: A case of shutdown policy in South Korea. Telematics and Informatics.

[bb0285] Lee Y., Park S. (2025, June 16). Conference Proceedings of DiGRA 2025: Games at the Crossroads. DiGRA 2025.

[bb0290] Lemmens J.S. (2022). Play or pay to win: Loot boxes and gaming disorder in FIFA ultimate team. Telematics and Informatics Reports.

[bb0295] Li W., Mills D., Nower L. (2019). The relationship of loot box purchases to problem video gaming and problem gambling. Addictive Behaviors.

[bb0300] McEvoy S. (2024, January 4). https://www.gamesindustry.biz/nexon-fined-89m-for-misleading-maplestory-players.

[bb0305] McMahon C., Wilska T.-A., Nyrhinen J. (2025). Young people in digital environments.

[bb0310] Mills S., Ash J., Gordon R. (2024). Children and young people’s experiences and understandings of gambling-style systems in digital games: Loot boxes, popular culture, and changing childhoods. Annals of the American Association of Geographers.

[bb0315] Mills S., Ash J., Gordon R. (2024). Digital geographies of home: Parenting practices in the space between gaming and gambling. Children’s Geographies.

[bb0320] Ministry of Higher Education and Science (Denmark) (2014). Danish Code of Conduct for Research Integrity. https://ufm.dk/en/publications/2014/the-danish-code-of-conduct-for-research-integrity.

[bb0325] Montiel I., Basterra-González A., Machimbarrena J.M., Ortega-Barón J., González-Cabrera J. (2022). Loot box engagement: A scoping review of primary studies on prevalence and association with problematic gaming and gambling. PLoS One.

[bb0330] Moon Active (2023). Coin Master > Cards & Chests > Probability. Coin Master.

[bb0335] Moshirnia A. (2018). Precious and worthless: A comparative perspective on loot boxes and gambling. Minnesota Journal of Law, Science & Technology.

[bb0340] Newzoo (2024). https://newzoo.com/resources/rankings/top-10-countries-by-game-revenues.

[bb0345] Nicklin L.L., Spicer S.G., Close J., Parke J., Smith O., Raymen T., Lloyd J. (2021). “It’s the attraction of winning that draws you in”—A qualitative investigation of reasons and facilitators for videogame loot box engagement in UK gamers. Journal of Clinical Medicine.

[bb0350] Nieborg D.B., Young C.J., Joseph D. (2020). App imperialism: The political economy of the Canadian App Store. Social Media + Society.

[bb0355] Nielsen R.K.L., Grabarczyk P. (2019). Are loot boxes gambling? Random reward mechanisms in video games. Transactions of the Digital Games Research Association.

[bb0360] Palmer L., Brooks G.A., Clark L. (2025). A longitudinal replication study testing migration from video game loot boxes to gambling in British Columbia, Canada. BMC Psychology.

[bb0365] Park S., Denoo M., Grosemans E., Petrovskaya E., Jin Y., Xiao L.Y. (2023). Proceedings of the 26th International Academic Mindtrek Conference.

[bb0370] Partis D. (2022, May 30). https://www.gamesindustry.biz/articles/2022-05-30-diablo-immortal-wont-launch-in-two-countries-due-to-loot-box-legislation.

[bb0375] Petrovskaya E., Deterding S., Zendle D. (2022). Proceedings of the 2022 CHI conference on human factors in computing systems.

[bb0380] Petrovskaya E., Zendle D. (2023). The relationship between psycho-environmental characteristics and wellbeing in non-spending players of certain mobile games. Royal Society Open Science.

[bb0385] Rossi R., Wheaton J.J., Moxey M., Moradipour S., Tozzi E., Singer J. (2024, September 24). https://hdl.handle.net/1983/3312a56d-d20e-429d-9e0a-17a80d522601.

[bb0390] Rousseau J. (2024, February 1). https://www.gamesindustry.biz/dataai-genshin-impact-hits-5bn-faster-than-any-other-mobile-title.

[bb0395] Rowland M. (2023, September 23). https://minister.infrastructure.gov.au/rowland/interview/transcript-press-conference-sydney.

[bb0400] Russell G.E.H., Sterner G.E., Kaye M.P., Ahlgren M.B. (2024). Online gambling in Pennsylvania. International Gambling Studies.

[bb0405] Sato Y., Brückner S., Michael Splichal J., Waragai I., Kurabayashi S. (2024). Cross-regional analysis of RRM design and implementation in mobile games by developers in China, the EU, Japan, and the USA. Entertainment Computing.

[bb0410] Sinclair B. (2023, May 23). https://www.gamesindustry.biz/activision-blizzard-plaion-fined-for-not-disclosing-loot-boxes.

[bb0415] Spicer S.G., Nicklin L.L., Uther M., Lloyd J., Lloyd H., Close J. (2022). Loot boxes, problem gambling and problem video gaming: A systematic review and meta-synthesis. New Media & Society.

[bb0420] Thorhauge A.M., Nielsen R.K.L. (2021). Epic, Steam, and the role of skin-betting in game (platform) economies. Journal of Consumer Culture.

[bb0425] UK Gambling Commission (2019). Young people and gambling survey 2019: A research study among 11-16 year olds in Great Britain. https://web.archive.org/web/20210129123612/https://www.gamblingcommission.gov.uk/PDF/Young-People-Gambling-Report-2019.pdf.

[bb0430] Ukie (UK Interactive Entertainment) (2023, July 18). New principles and guidance on paid loot boxes. https://web.archive.org/web/20250414060741/https://ukie.org.uk/loot-boxes.

[bb0435] Wen J., Zhang Z., Tran T.M., Mu L., Rahman T., Jin H. (2024). Folk models of loot boxes in video games. Proceedings of the ACM on Human-Computer Interaction.

[bb0440] Woods O. (2022). The economy of time, the rationalisation of resources: Discipline, desire and deferred value in the playing of Gacha games. Games and Culture.

[bb0445] Xiao L.Y. (2021). Conceptualising the loot box transaction as a gamble between the purchasing player and the video game company. International Journal of Mental Health and Addiction.

[bb0450] Xiao L.Y. (2021). Regulating loot boxes as gambling? Towards a combined legal and self-regulatory consumer protection approach. Interactive Entertainment Law Review.

[bb0455] Xiao L.Y. (2022). Drafting video game loot box regulation for dummies: A Chinese lesson. Information & Communications Technology Law.

[bb0460] Xiao L.Y. (2022). Reserve your judgment on “draconian” Chinese video gaming restrictions on children. Journal of Behavioral Addictions.

[bb0465] Xiao L.Y. (2023). Beneath the label: Unsatisfactory compliance with ESRB, PEGI, and IARC industry self-regulation requiring loot box presence warning labels by video game companies. Royal Society Open Science.

[bb0470] Xiao L.Y. (2023). Breaking ban: Belgium’s ineffective gambling law regulation of video game loot boxes. Collabra: Psychology.

[bb0475] Xiao L.Y. (2023). Opening the compliance and enforcement loot box: A retrospective on some practice and policy impacts achieved through academic research. Societal Impacts.

[bb0480] Xiao L.Y. (2023). Shopping around for loot box presence warning labels: Unsatisfactory compliance on Epic, Nintendo, Sony, and Microsoft platforms. ACM Games: Research and Practice.

[bb0485] Xiao L.Y. (2024).

[bb0490] Xiao L.Y. (2024). Loot box state of play 2023: Law, regulation, policy, and enforcement around the world. Gaming Law Review.

[bb0495] Xiao L.Y. (2024).

[bb0500] Xiao L.Y. (2025). Failing to protect the online consumer: Poor compliance with Dutch loot box and video game consumer protection guidelines. International Journal of Law and Information Technology.

[bb0505] Xiao L.Y. (2025). Illegal loot box advertising on social media? An empirical study using the Meta and TikTok ad transparency repositories. Computer Law & Security Review.

[bb0510] Xiao L.Y., Brooks G. (2024).

[bb0515] Xiao L.Y., Declerck P. (2023). Paid video game loot boxes are not gambling under Dutch gambling regulation? Shifting the goalpost in Electronic Arts v. Kansspelautoriteit. Gaming Law Review.

[bb0520] Xiao L.Y., Deery C. (2025).

[bb0525] Xiao L.Y., Deery C., Petrovskaya E., Park S., Newall P. (2025). Widespread illegal video game advertising in the UK and South Korea: Many adverts not disclosing loot box presence found using Meta’s ad repository. Journal of Behavioral Addictions.

[bb0530] Xiao L.Y., Fraser T.C., Newall P.W.S. (2023). Opening pandora’s loot box: Weak links between gambling and loot box expenditure in China, and player opinions on probability disclosures and pity-timers. Journal of Gambling Studies.

[bb0535] Xiao L.Y., Fraser T.C., Nielsen R.K.L., Newall P.W.S. (2024). Loot boxes, gambling-related risk factors, and mental health in mainland China: A large-scale survey. Addictive Behaviors.

[bb0540] Xiao L.Y., Henderson L.L., Newall P. (2023). What are the odds? Lower compliance with Western loot box probability disclosure industry self-regulation than Chinese legal regulation. PLoS One.

[bb0545] Xiao L.Y., Henderson L.L., Newall P.W.S. (2022). Loot boxes are more prevalent in United Kingdom video games than previously considered: Updating Zendle et al. (2020). Addiction.

[bb0550] Xiao L.Y., Henderson L.L., Nielsen R.K.L., Grabarczyk P., Newall P.W.S., Lee N. (2021). Encyclopedia of Computer Graphics and Games.

[bb0555] Xiao L.Y., Henderson L.L., Nielsen R.K.L., Newall P.W.S. (2022). Regulating gambling-like video game loot boxes: A public health framework comparing industry self-regulation, existing national legal approaches, and other potential approaches. Current Addiction Reports.

[bb0560] Xiao L.Y., Henderson L.L., Yang Y., Newall P.W.S. (2024). Gaming the system: Suboptimal compliance with loot box probability disclosure regulations in China. Behavioural Public Policy.

[bb0565] Xiao L.Y., Lund M. (2025). Non-compliance with and non-enforcement of UK loot box industry self-regulation on the Apple App Store: A longitudinal study on poor implementation. Royal Society Open Science.

[bb0570] Xiao L.Y., Petrovskaya E., Khoo N., Denoo M., Roberts A. (2025).

[bb0575] Yang Q., Wang H., Wu H., Li W., Zhang Y., Yao Y., Yuan X., Chen C., Wang Y., Zhong Y., Wang W., Zhang M., Yang Y., Liu H., Zhang K. (2023). Effect of online game policy on smartphone game play time, addiction, and emotion in rural adolescents of China. BMC Psychiatry.

[bb0580] Yokomitsu K., Irie T., Shinkawa H., Tanaka M. (2021). Characteristics of gamers who purchase loot box: A systematic literature review. Current Addiction Reports.

[bb0585] Zendle D., Cairns P. (2018). Video game loot boxes are linked to problem gambling: Results of a large-scale survey. PLoS One.

[bb0590] Zendle D., Cairns P., Meyer R., Waters S., Ballou N. (2022). If everything is a loot box, nothing is: Response to Xiao et al. Addiction.

[bb0595] Zendle D., Flick C., Deterding S., Cutting J., Gordon-Petrovskaya E., Drachen A. (2023). The many faces of monetisation: Understanding the diversity and extremity of player spending in mobile games via massive-scale transactional analysis. Games: Research and Practice.

[bb0600] Zendle D., Flick C., Gordon-Petrovskaya E., Ballou N., Xiao L.Y., Drachen A. (2023). No evidence that Chinese playtime mandates reduced heavy gaming in one segment of the video games industry. Nature Human Behaviour.

[bb0605] Zendle D., Meyer R., Cairns P., Waters S., Ballou N. (2020). The prevalence of loot boxes in mobile and desktop games. Addiction.

[bb0610] Zendle D., Meyer R., Over H. (2019). Adolescents and loot boxes: Links with problem gambling and motivations for purchase. Royal Society Open Science.

[bb0615] Zhou X., Liao M., Gorowska M., Chen X., Li Y. (2024). Compliance and alternative behaviors of heavy gamers in adolescents to Chinese online gaming restriction policy. Journal of Behavioral Addictions.

[bb0620] 게임물관리위원회 [Game Rating and Administration Committee] (GRAC) (South Korea) (2024). Rating guide. http://www.grac.or.kr/english/enforcement/ratingguide.aspx.

[bb0625] 게임물관리위원회 [Game Rating and Administration Committee] (GRAC) (South Korea) (2024, March 22). 확률정보공개 관련 사업자 FAQ 게시 안내 [Information on posting FAQs for businesses related to probability information disclosure]. 게임물관리위원회. https://www.grac.or.kr/Board/Inform.aspx?searchtype=004&type=view&bno=826&searchtext=.

[bb0630] 게임물관리위원회 [Game Rating and Administration Committee] (GRAC) (South Korea) (2024, April 6). Age rating symbol [archived by the wayback machine on 6 April 2024]. https://web.archive.org/web/20240406224132/https://www.grac.or.kr/english/enforcement/ageratingsymbol.aspx.

[bb0635] 게임물관리위원회 [Game Rating and Administration Committee] (GRAC) (South Korea) (2024, July 17). Age rating symbol. https://www.grac.or.kr/english/enforcement/ageratingsymbol.aspx.

[bb0640] 김 [Kim], 미희 [Mi-hee] (2024, July 3). 확률 공개 시행 100일, 적발된 게임 266개 [100 days after probability disclosure implementation, 266 games detected]. 게임메카 [GameMeca]. https://www.gamemeca.com/view.php?gid=1750633.

[bb0645] 넥슨 [Nexon] (2023, December 22). 게임산업법 청소년 정의 변경에 따른 넥슨 서비스 영향성 안내 [Guidance on the impact of Nexon services due to changes in the definition of youth in the Game Industry Act]. https://notice.nexon.com/Notice/NoticeView?sn=143040&maskgamecode=65536.

[bb0650] 대한민국 국회 [National Assembly of the Republic of Korea] (2020, December 15). [2106496] 게임산업진흥에 관한 법률 전부개정법률안(이상헌의원 등 17인) [[2106496] A bill to amend the entirety of the Game Industry Promotion Act (17 members including Lee Sang-heon)]. https://likms.assembly.go.kr/bill/billDetail.do?billId=PRC_E2I0I1I2R1N4M1C5J5H2O3E3R4M1O3.

[bb0655] 문화체육관광부 [Ministry of Culture, Sports and Tourism] (South Korea) (2024, January 9). 게임산업진흥에 관한 법률 시행령 [Enforcement Decree of the Game Industry Promotion Act] (as amended by Presidential Decree No. 34114 of 9 January 2024). https://www.law.go.kr/%EB%B2%95%EB%A0%B9/%EA%B2%8C%EC%9E%84%EC%82%B0%EC%97%85%EC%A7%84%ED%9D%A5%EC%97%90%EA%B4%80%ED%95%9C%EB%B2%95%EB%A5%A0%EC%8B%9C%ED%96%89%EB%A0%B9.

[bb0660] 문화체육관광부 [Ministry of Culture, Sports and Tourism] (South Korea) (2024, January 9). 별표 3의2: 확률형 아이템 공급 확률정보 등의 표시내용 및 표시방법(제19조의2제3항 관련) [Appendix 3–2: Display contents and display method of probability information on supply of probability type items (related to Article 19–2, Paragraph 3)]. https://www.law.go.kr/%EB%B2%95%EB%A0%B9%EB%B3%84%ED%91%9C%EC%84%9C%EC%8B%9D/(%EA%B2%8C%EC%9E%84%EC%82%B0%EC%97%85%EC%A7%84%ED%9D%A5%EC%97%90%20%EA%B4%80%ED%95%9C%20%EB%B2%95%EB%A5%A0%20%EC%8B%9C%ED%96%89%EB%A0%B9,20240322,%EB%B3%84%ED%91%9C3%EC%9D%982).

[bb0665] 문화체육관광부 [Ministry of Culture, Sports and Tourism] (South Korea) (2024, February 19). 확률형 아이템 확률정보공개 관련 해설서 배포 안내 [Guideline on the Disclosure of Probability Information for Probabilistic Item] [Original Korean Version]. https://www.mcst.go.kr/kor/s_notice/notice/noticeView.jsp?pSeq=17856.

[bb0670] 문화체육관광부 [Ministry of Culture, Sports and Tourism] (South Korea) (2024, March 15). 확률형 아이템 확률정보공개 관련 해설서 배포 안내 [Guideline on the Disclosure of Probability Information for Probabilistic Item] [Official English Translation]. https://www.mcst.go.kr/kor/s_notice/notice/noticeView.jsp?pSeq=17910.

[bb0675] 한국게임산업협회 [Korea Association of Game Industry; K-GAMES] (2017, February 15). 건강한 게임문화 조성을 위한 자율규제 강령 [Self-regulatory code for creating a healthy game culture] (enacted 15 February 2017). http://www.gsok.or.kr/regulations-on-self-regulation/?pageid=2&mod=document&uid=79.

[bb0680] 한국게임산업협회 [Korea Association of Game Industry; K-GAMES] (2018). 건강한 게임문화 조성을 위한 자율규제 시행기준 [Criteria on Implementation of Self-regulation for Healthy Game Culture] (revised 1 July 2018). http://www.gsok.or.kr/regulations-on-self-regulation/?uid=89&mod=document&pageid=1.

[bb0685] 한국게임산업협회 [Korea Association of Game Industry; K-GAMES] (2020, June 22). 확률형아이템 자율규제 미준수 게임물 19차 공표 [The 19th Announcement of Games which are Non-Compliant with Self-Regulatory Probabilities Disclosure Obligations]. http://www.gsok.or.kr/wp-content/uploads/2020/06/%EB%B3%B4%EB%8F%84%EC%9E%90%EB%A3%8C%ED%99%95%EB%A5%A0%ED%98%95%EC%95%84%EC%9D%B4%ED%85%9C-%EC%9E%90%EC%9C%A8%EA%B7%9C%EC%A0%9C-%EB%AF%B8%EC%A4%80%EC%88%98-%EA%B2%8C%EC%9E%84%EB%AC%BC-19%EC%B0%A8-%EA%B3%B5%ED%91%9C.pdf.

[bb0690] 한국게임산업협회 [Korea Association of Game Industry; K-GAMES] (2020, October 19). 확률형아이템 자율규제 미준수 게임물 23차 공표 [The 23rd Announcement of Games which are Non-Compliant with Self-Regulatory Probability Disclosure Obligations]. http://www.gsok.or.kr/wp-content/uploads/2020/06/%EB%B3%B4%EB%8F%84%EC%9E%90%EB%A3%8C%ED%99%95%EB%A5%A0%ED%98%95%EC%95%84%EC%9D%B4%ED%85%9C-%EC%9E%90%EC%9C%A8%EA%B7%9C%EC%A0%9C-%EB%AF%B8%EC%A4%80%EC%88%98-%EA%B2%8C%EC%9E%84%EB%AC%BC-19%EC%B0%A8-%EA%B3%B5%ED%91%9C.pdf.

[bb0695] 한국게임산업협회 [Korea Association of Game Industry; K-GAMES] (2021, August 17). 확률형 아이템 자율규제 미준수 게임물 33차 공표 [The 33rd Announcement of Games which are Non-Compliant with Self-Regulatory Probability Disclosure Obligations]. 한국게임정책자율기구 GSOK. http://www.gsok.or.kr/?kboard_content_redirect=1603.

[bb0700] 한국게임산업협회 [Korea Association of Game Industry; K-GAMES] (2024, January 21). 한국게임정책자율기구, 2023년 12월 확률형 콘텐츠 확률공개 미준수 게임물 리스트 공표 [GSOK announces list of games that do not comply with probability disclosure of probability-type content in December 2023]. http://www.gsok.or.kr/?kboard_content_redirect=2975.

[bb0705] 国家新闻出版署 [National Press and Publication Administration (PRC)] (2021, August 30). 国家新闻出版署关于进一步严格管理切实防止未成年人沉迷网络游戏的通知 [Notice of the National Press and Publication Administration on Further Strictly Regulating and Effectively Preventing Online Video Gaming Addiction in Minors] 国新出发〔2021〕14号. https://web.archive.org/web/20210830120201/http://www.nppa.gov.cn/nppa/contents/279/98792.shtml.

[bb0710] 文化部 [Ministry of Culture] (PRC) (2016, December 1). ⽂化部关于规范⽹络游戏运营加强事中事后监管⼯作的通知 [Notice of the Ministry of Culture on Regulating the Operation of Online Games and Strengthening Concurrent and Ex-Post Supervisions] 文市发〔2016〕32号. https://web.archive.org/web/20171220060527/http://www.mcprc.gov.cn:80/whzx/bnsjdt/whscs/201612/t20161205_464422.html.

[bb0715] 消費者保護處 [Consumer Protection Office] (Taiwan) (2022, December 29). 網路連線遊戲服務定型化契約應記載及不得記載事項 [Matters that should be recorded and should not be recorded in the standardised contracts of online game services] (as amended on 10 August 2022, effective 1 January 2023). 行政院 [Executive Yuan]; 2.16.886.101.20003. https://www.ey.gov.tw/Page/DFB720D019CCCB0A/964028ea-f1f6-4383-9c78-f7d0606086f3.

[bb0720] 行政院消費者保護會消費者保護處 [Consumer Protection Office, Consumer Protection Committee, Executive Yuan] (Taiwan) (2022, July 15). 公布轉蛋中獎機率 保障遊戲玩家權益 [Disclosing Loot Box Odds to Protect Gamers' Interests]. 2.16.886.101.20003. https://cpc.ey.gov.tw/Page/6C059838CA9744A8/adc0330c-bd72-416b-9ecf-08e6a9d339ec.

